# Predicting Mental and Neurological Illnesses Based on Cerebellar Normative Features

**DOI:** 10.1016/j.bpsgos.2025.100541

**Published:** 2025-05-28

**Authors:** Milin Kim, Nitin Sharma, Esten H. Leonardsen, Saige Rutherford, Geir Selbæk, Karin Persson, Nils Eiel Steen, Olav B. Smeland, Torill Ueland, Geneviève Richard, Aikaterina Manoli, Sofie L. Valk, Dag Alnæs, Christian F. Beckman, Andre F. Marquand, Ole A. Andreassen, Lars T. Westlye, Thomas Wolfers, Torgeir Moberget

**Affiliations:** a Centre for Precision Psychiatry, Division of Mental Health and Addiction, University of Oslo and Oslo University Hospital, Oslo, Norway; b Department of Psychology, University of Oslo, Oslo, Norway; c Department of Psychiatry and Psychotherapy, University of Tübingen, Tübingen, Germany; d German Center for Mental Health, Tübingen, Germany; e Department of Cognitive Neuroscience, Radboud University Medical Centre, Nijmegen, the Netherlands; f Donders Institute, Radboud University, Nijmegen, the Netherlands; g Department of Psychiatry, University of Michigan, Ann Arbor, Michigan; h Department of Geriatric Medicine, Oslo University Hospital, Oslo, Norway; i The Norwegian National Centre for Ageing and Health, Vestfold Hospital Trust, Tønsberg, Norway; j Institute for Clinical Medicine, University of Oslo, Oslo, Norway; k Section for Clinical Psychosis Research, Division of Mental Health and Addiction, Oslo University Hospital, Oslo, Norway; l Division of Mental Health and Substance Abuse, Diakonhjemmet Hospital, Oslo, Norway; m Otto Hahn Research Group for Cognitive Neurogenetics, Max Planck Institute for Human Cognitive and Brain Sciences, Leipzig, Germany; n Institute of Neurosciences and Medicine (INM-7), Research Centre Jülich, Jülich, Germany; o Institute of Systems Neuroscience, Heinrich-Heine-Universität Düsseldorf, Düsseldorf, Germany; p Centre for Functional MRI of the Brain, Nuffield Department of Clinical Neurosciences, Wellcome Centre for Integrative Neuroimaging, University of Oxford, Oxford, United Kingdom; q Department of Neuroimaging, Center of Neuroimaging Sciences, Institute of Psychiatry, King’s College London, London, United Kingdom; r KG Jebsen Centre for Neurodevelopmental Disorders, University of Oslo, Oslo, Norway; s Department of Behavioral Science, School of Health Sciences, Oslo Metropolitan University, Oslo, Norway; t Department of Psychology, Pedagogy and Law, School of Health Sciences, Kristiania University College, Oslo, Norway

**Keywords:** Cerebellum, Machine learning, Magnetic resonance imaging, Mental illnesses, Neurological diseases, Normative modeling

## Abstract

**Background:**

Mental and neurological conditions have been linked to structural brain variations. However, aside from dementia, the value of brain structural characteristics derived from brain scans for prediction is relatively low. One reason for this limitation is the clinical and biological heterogeneity inherent to such conditions. Recent studies have implicated aberrations in the cerebellum, a relatively understudied brain region, in these clinical conditions.

**Methods:**

Here, we used machine learning to test the value of individual deviations from normative cerebellar development across the lifespan (based on trained data from >27,000 participants) for prediction of autism spectrum disorder (ASD) (*n* = 317), bipolar disorder (*n* = 238), schizophrenia (SZ) (*n* = 195), mild cognitive impairment (*n* = 122), and Alzheimer's disease (*n* = 116); individuals without diagnoses were matched to the clinical cohorts. We applied several atlases and derived median, variance, and percentages of extreme deviations within each region of interest.

**Results:**

The results show that lobular and voxelwise cerebellar data can be used to discriminate reference samples from individuals with ASD and SZ with moderate accuracy (the area under the receiver operating characteristic curves ranged from 0.56 to 0.65). Contributions to these predictive models originated from both anterior and posterior regions of the cerebellum.

**Conclusions:**

Our study highlights the utility of cerebellar normative modeling in predicting ASD and SZ, aided by 4 cerebellar atlases that enhanced the interpretability of the findings.

## Plain Language Summary

Recent research has shown that the cerebellum plays a role in various clinical conditions. In this study, we explored the ability to predict 5 mental and neurological conditions using features derived from the cerebellum. By utilizing machine learning and combining 4 existing cerebellar maps, the analysis revealed moderate prediction of autism spectrum disorder (ASD) and schizophrenia (SZ), with both anterior and posterior regions of cerebellar regions providing insights into these conditions.

Clinical heterogeneity and complex pathobiological mechanisms impede the discovery of reliable biomarkers for many neurological and especially psychiatric disorders, thereby complicating precise clinical decision making and treatments. Over the last 2 decades, there has been a trend in the development of neuroimaging-based tools and machine learning for prognosis and diagnosis of psychiatric disorders (1,2) and neurological illnesses (3). Neuroimaging-based prediction studies of autism spectrum disorder (ASD), bipolar disorder (BD), and schizophrenia (SZ) have reported a wide range of accuracies, underscoring the limitations associated with small samples, including poor generalization performance (4,5). Notably, prediction studies on dementia have shown greater promise for clinical usage in both Alzheimer’s disease (AD) (3) and mild cognitive impairment (MCI).

Notably, most of these prediction studies (4, 5, 6) have focused on cerebral features, perhaps reflecting a corticocentric bias in the literature (7). Nonetheless, disruptions in the cerebellum have been hypothesized to contribute to early cognitive disturbances (8) and various clinical conditions, such as childhood psychiatric symptoms (9), AD (10), SZ (11), and ASD (12, 13, 14). Using a normative modeling approach, we recently demonstrated significant deviations from normal cerebellar developmental across the lifespan in individuals with mental and neurodegenerative illnesses including SZ and AD (15). The deviation refers to instances where an individual falls outside the typical range. However, recent literature indicates that there are no significant differences in the cerebellum in ASD (16), a condition characterized by repetitive patterns in behaviors, restricted interests, and difficulties in social interaction and communication (17). This may be attributed to the presence of both positive and negative deviations in cerebellar characteristics (15). In contrast, several studies (11,18) of SZ indicate a reduction in cerebellar volume, particularly in regions associated with perception, language comprehension, and cognitive functions. Patient studies have shown that abnormalities in the cerebellum can exert a significant influence on motor, cognitive, and emotional functions (19, 20, 21); however, there has been little exploration of the role of the cerebellum in predicting and classifying mental and neurological illnesses. While these individual-level deviations revealed substantial cerebellar heterogeneity among individuals with the same disorder, the value of these cerebellar features with respect to classifying these disorders remains uncertain.

In this study, we addressed this gap by performing a set of predictions of ASD, MCI, AD, BD, and SZ using magnetic resonance imaging (MRI)–based cerebellar features and cross-validated machine learning classifiers. We applied lobular and voxelwise normative models (15) and aggregated the median, variance, and percentage of extreme deviations across atlases (22,23). The features, specifically the percentage of extreme deviation, median, and variance for each of the different cerebellar atlas regions, illuminate the model performance in terms of prediction of the disorders and diseases, given different parcellations and features. Finally, for models that were able to meaningfully differentiate between patients and healthy control participants, we identified the cerebellar regions that contributed most to the prediction.

## Methods and Materials

### Sample

**Figure 1:**
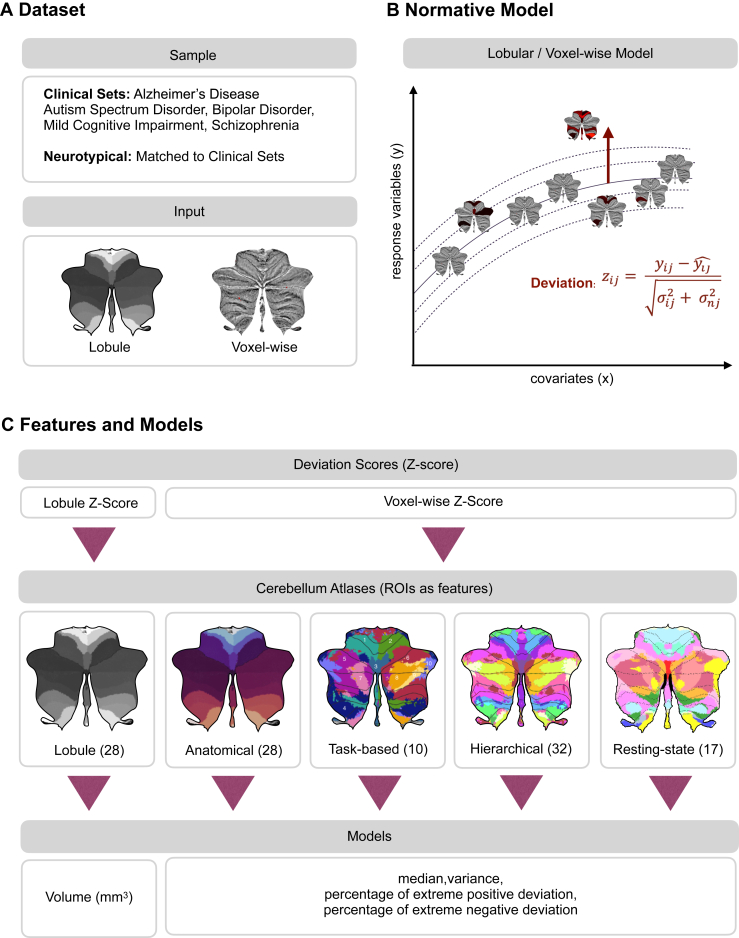
Overview of predicting mental and neurological illnesses. **(A)** The study included 5 clinical datasets: Alzheimer’s disease, autism spectrum disorder, bipolar disorder, mild cognitive impairment, and schizophrenia. The input data, together with the samples, consist of both lobular and voxelwise data. **(B)** Individuals without a diagnosis were divided into training and test sets to evaluate the cerebellar normative models. The deviation score (*z* score) measures how much an individual deviates from the norm represented by the estimated population model. **(C)** The analysis utilized the deviation scores derived from the cerebellar lobular and voxelwise normative models. Lobular *z* scores consist of 28 lobular volumes. For voxelwise, deviation scores overlaid onto existing cerebellar atlases including anatomical, task-based, hierarchical, and resting-state parcellations. This process calculated median, variance, and percentage of extreme positive and negative deviation for each atlas’ regions of interest (ROIs). Logistic regression was used for each atlas to assess the predictive value of features across all ROIs.

The study sample consisted of individuals from the cerebellar lifespan normative model (15), where individuals without a diagnosis were split into a training set (*n* = 27,117; 54% female), a test set (*n* = 26,985; 53% females), and a clinical set (*n* = 1757; 30% females) (Figure 1A and Table S2). Individuals without diagnoses were matched to the clinical datasets of patients with AD, ASD, BD, MCI, and SZ (Table 1) using nearest-neighbor matching based on exact matches of sex and scanning site with age as implemented in *MatchIt* (24). The clinical datasets were obtained from the ABIDE I and II (Autism Brain Imaging Data Exchange I and II), ADNI (Alzheimer’s Disease Neuroimaging Initiative), AIBL (The Australian Imaging, Biomarkers and Lifestyle Flagship Study), DEMGEN (Norwegian Dementia Genetics Network), and TOP (Thematically Organized Psychosis) cohorts. Information about each cohort and studies can be found in the corresponding publications (Table S1). If participants were scanned at several time points, only baseline scans were chosen for this study. Individuals who withdrew from the studies or lacked essential demographic information and T1-weighted MRI data were excluded from the analyses.

**Table 1:** Matched Sample Description and Demographics

Sample	Participants	Scanners	Age, Years	Sex, Female/Male
Matched HC				
Alzheimer’s Disease	116	13	71.72 (7.12)	55%/45%
ASD	317	25	15.96 (7.45)	17%/83%
Bipolar Disorder	238	3	33.06 (10.50)	55%/45%
Mild Cognitive Impairment	122	3	65.62 (9.91)	42%/58%
Schizophrenia	195	3	30.13 (8.15)	41%/59%

Clinical				
Alzheimer’s Disease	116	13	73.11 (7.60)	55%/45%
ASD	317	25	12.35 (4.42)	17%/83%
Bipolar Disorder	238	3	31.61 (11.40)	55%/45%
Mild Cognitive Impairment	122	3	67.25 (9.27)	42%/58%
Schizophrenia	195	3	28.29 (9.45)	41%/59%

Values are presented as *n*, mean (SD), or %.

ASD, autism spectrum disorder; HC, healthy control participant.

### Lobular-Level Processing

The T1-weighted images were skull stripped using the FreeSurfer version 5.3 auto-recon pipeline (25) and reoriented to the standard FSL orientation using the *fslreorient2std* (26). Linear registration was performed using *flirt* (27), which utilized linear interpolation (with 6 degrees of freedom) and the default 1 mm FSL template (version 6.0). The borders were cropped at coordinates (6:173, 2:214, 0:160) to minimize their size without removing brain tissue. Finally, the voxel intensity values of all brain images were normalized to the range of (0, 1), adjusting the intensity values of each voxel to a standardized scale.

To segment the cerebellum, we utilized the Automatic Cerebellum Anatomical Parcellation Using U-Net with Locally Constrained Optimization (ACAPULCO) algorithm (28), a part of the Enhancing Neuro Imaging Genetics through Meta Analysis (ENIGMA) Cerebellum Volumetric Pipeline, which is a cerebellum parcellation algorithm based on convolutional neural networks. This algorithm delivers fast and precise quantitative in vivo regional assessment of the cerebellum. As part of the algorithm, the images were corrected for inhomogeneity by the N4 correction method (29) and registered to the 1-mm isotropic ICBM 2009c template in Montreal Neurological Institute (MNI) space using the Advanced Normalization Tools registration suite (30). The ACAPULCO algorithm is based on 15 expert manual delineations of an adult cohort (31). It achieves per-voxel labeling and uses postprocessing of the parcellation to correct for mislabeling and accurate segmentation. ACAPULCO segments the cerebellum into 28 cerebellar lobules and computes the volume (mm^3^) for each lobule. These regions include bilateral lobules I–VI; crus I and II; lobules VIIB, VIIIA, VIIIB, and IX–X; vermis VI, VII, VIII, IX, and X; and the corpus medullare. To ensure data quality, participants with extreme outliers (2.698 SD above or below the mean) (32) in more than 2 lobules based on automated quality control measures were excluded. We set the threshold at 2 lobules because the differences between 1 and 2 lobules were not significant (see Supplemental Methods for detailed information on quality control).

### Voxel-Level Processing

We used Spatially Unbiased Infratentorial Toolbox (SUIT) version 3.4 (33) to segment cerebellar gray and white matter voxel-based morphometry maps. SUIT leverages the outputs from ACAPULCO, an MNI-aligned T1 image (33,34), and an average mask derived from a randomly selected group of 300 individuals without a diagnosis. After segmentation, the gray matter maps were standardized for comparison by aligning them to SUIT space through Jacobian modulation, ensuring that each voxel reflected its proportional alignment to the original volume.

### Normative Modeling

Normative modeling (35), similar to pediatric growth charts, addresses the limitation of case-control studies by mapping developmental and aging trajectories across the lifespan, thereby preserving individual-level inference in reference to large populations (36, 37, 38). Widely applied in clinical research (38, 39, 40, 41, 42, 43), it helps detect extreme deviations in individuals. Here, we used this approach to normalize clinical groups against the population reference, accounting for age, sex, and site effects. We used a publicly available cerebellar normative model based on >27,000 individuals without diagnoses (15). The cerebellar normative model was developed using a large reference sample that spans ages 3 to 85 years. This broad age range allows us to map individual deviations from established norms within clinical cohorts, including young participants. The use of this extensive reference sample provides a basis for normalization and confound removal with respect to age, sex, and site. This normative model includes cerebellar lobules and voxelwise intensities, which differ across tissues (e.g., gray matter, white matter, cerebrospinal fluid) based on their proton density and relaxation properties, while adjusting for sex, age, and scanning site (Figure 1B).

To analyze the data, we utilized Bayesian linear regression with the likelihood warping method (44), incorporating the sinarcsinsh transformation (45,46), to handle nonlinear basis functions and non-Gaussian predictive distributions for large datasets (45). Scanning site was accounted for as a fixed effect (47,48). The normative model provides point estimates and evaluation metrics such as explained variance, mean squared log loss, skew, and kurtosis (46). These evaluation metrics were calculated in the test set, which did not include clinical cohorts. Extreme deviations were defined as |*z*| > 1.96, corresponding to the most extreme 5% of cases in both directions in the reference cohort.

### Feature Engineering

First, lobular normative models were used to derive deviation scores of volume for the 28 cerebellar lobules (Table S3). Second, voxelwise normative models were utilized to map deviation profiles of gray matter onto existing atlases (see Supplemental Methods for statistics and reproducibility). Four existing atlases were selected: 28 regions of interest (ROIs) from cerebellar anatomical atlas, 10 ROIs from a multidomain task battery (23), 32 ROIs from a hierarchical atlas (49), and 17 ROIs from resting-state connectivity (22,50) (see Atlases in Supplemental Methods and Figure S1 for labels). For each ROI delineated by these atlases, we computed 3 key statistics: the median, variance, and percentage of extreme deviations (Figure 1C). To quantify the percentage of extremes in deviation, we also calculated the proportion of voxelwise deviations that exceeded the established threshold of |*z*| > 1.96, denoting both extreme positive and negative deviations. This proportion was determined by dividing the count of such extreme deviations by the total voxel count within the corresponding ROI. Variance has previously been used to examine the structural heterogeneity among patients with SZ (51,52). Unlike percentage of extreme deviation (|*z*| > 1.96), which has been used in past normative studies (38,42,53), variance assesses the dispersion within the region, capturing the regionally heterogeneous spread within patients.

### Model Training and Evaluation

Machine learning models using logistic regression (LR) were used to build prediction models (Figure 1D). The LR model was trained on the provided dataset, utilizing a logistic function to optimize weights to best fit the data. In addition, results from the random forest (RF) algorithm from the scikit-learn library version 1.2.2 (54) and the eXtreme Gradient Boosting (XGBoost) library version 1.7.3 (55) can be found in Figure S4. RF is a nonparametric supervised learning method that addresses overfitting by combining decision trees into a single outcome, effectively balancing the bias-variance trade-off. XGBoost is an open-source library to implement advanced gradient boosting algorithms (55).

Features were engineered across 4 atlases as well as for the different cerebellar lobules, serving as input to the LR algorithm, with diagnoses with respect to healthy individuals and clinical groups, such as ASD, BD, SZ, MCI, and AD, as labels. We utilized LR to assess predictive performance, using deviations from normative models, specifically their median, variance, and percentage of extreme deviations, derived from existing atlases superimposed onto voxelwise cerebellar maps. To evaluate the model’s performance in the test set, we conducted a stratified 5-fold cross-validation and used the area under the receiver operating characteristic curve (AUROC) as the primary performance metric. The AUROC measures the ability of the model to distinguish between classes, in this case, accurately identifying individuals with or without the condition under study. A higher AUROC value, closer to 1, indicates better performance, signifying that the model has a higher probability of correctly classifying the outcomes. We also calculated precision, recall, sensitivity, specificity, balanced accuracy, and the area under the precision-recall curve.

### Permutation Testing

We used permutation testing to assess whether the AUROCs achieved by our model were different from chance-level performance. To achieve this, we shuffled the diagnosis labels randomly 1000 times for each permutation calculating an AUROC. For significance testing, the original AUROC was compared with the distribution of permuted AUROC values. If the original AUROC fell within the extreme ends of the permutation distribution (*p* < .05), it was considered statistically significant. We applied an identical approach for the lobular volume features. The comparison between models utilized an approach similar to that outlined in Figures S3 and S4, wherein the previously calculated shuffled AUROC values were used. We calculated the difference in true AUROC scores, as well as the AUROC differences from 1000 permuted datasets, between the 2 models. Subsequently, we compared the true score and the permuted scores to assess statistical significance.

### Feature Importance Ranking

We assessed feature importance based on LR coefficients to highlight their influence on the predictions. The coefficients from the model directly infer the relative importance of each feature, thus facilitating interpretation. The standardized magnitude of the coefficient indicates the strength of the effect that a feature has on the prediction, while the sign (positive or negative) indicates the direction of the effect.

## Results

We conducted a comprehensive analysis at the lobular and voxelwise level using a variety of models (Figure 1C). The voxelwise model calculations included variance, median, and percentage of deviations across 143,000 voxels, which were organized into 28 ROIs for the anatomical atlas, 10 ROIs for the task-based atlas, 32 ROIs for the hierarchical atlas, and 17 ROIs for the resting-state atlas.

**Figure 2:**
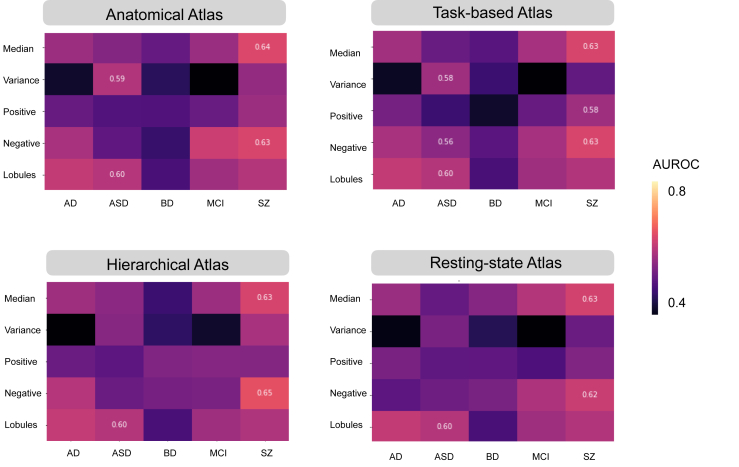
Cerebellar features moderately predict autism spectrum disorder (ASD) and schizophrenia (SZ). **(A)** Information from the anatomical (28 regions), task-based (10 regions), hierarchical (32 regions), or resting-state (17 regions) atlases are compiled into features that were used as predictors by the logistic regression model to make predictions. The area under the receiver operating characteristic curve (AUROC) serves as an important measure in evaluating the performance of a binary classifier, representing a trade-off between the classifier’s sensitivity (true positive rate) and specificity (true negative rate). The reliability and robustness of the AUROC were assessed by computing them over 1000 permutations, which aids in determining whether the classifier’s performance is statistically significant or due to random chance. **(B–D)** The values that survived multiple comparison are shown. AD, Alzheimer’s disease; BD, bipolar disorder; MCI, mild cognitive impairment.

Permutation testing revealed significant predictions for ASD and SZ (AUROC values ranging from 0.56 to 0.65), using various models based on deviations from the cerebellar normative model (Figure 2 and Table S4). Prediction performance for MCI, AD, and BD were not above chance levels. For SZ, the most predictive models were those centered around median and variance measures summarized within ROIs for the voxelwise models. In contrast, for ASD, models based on the lobular volumes and voxelwise variance within ROIs were found to be the most predictive. We explored 2 machine learning approaches, LR and RF, and observed no notable differences between models in terms of feature importance or performance (Figures S3 and S4). This indicates that despite using different methods and parcellation schemes, the core predictions remained robust, affirming the stability and reliability of our modeling approach. We report the linear and interpretable method in terms of features weighs in the main text.

**Figure 3:**
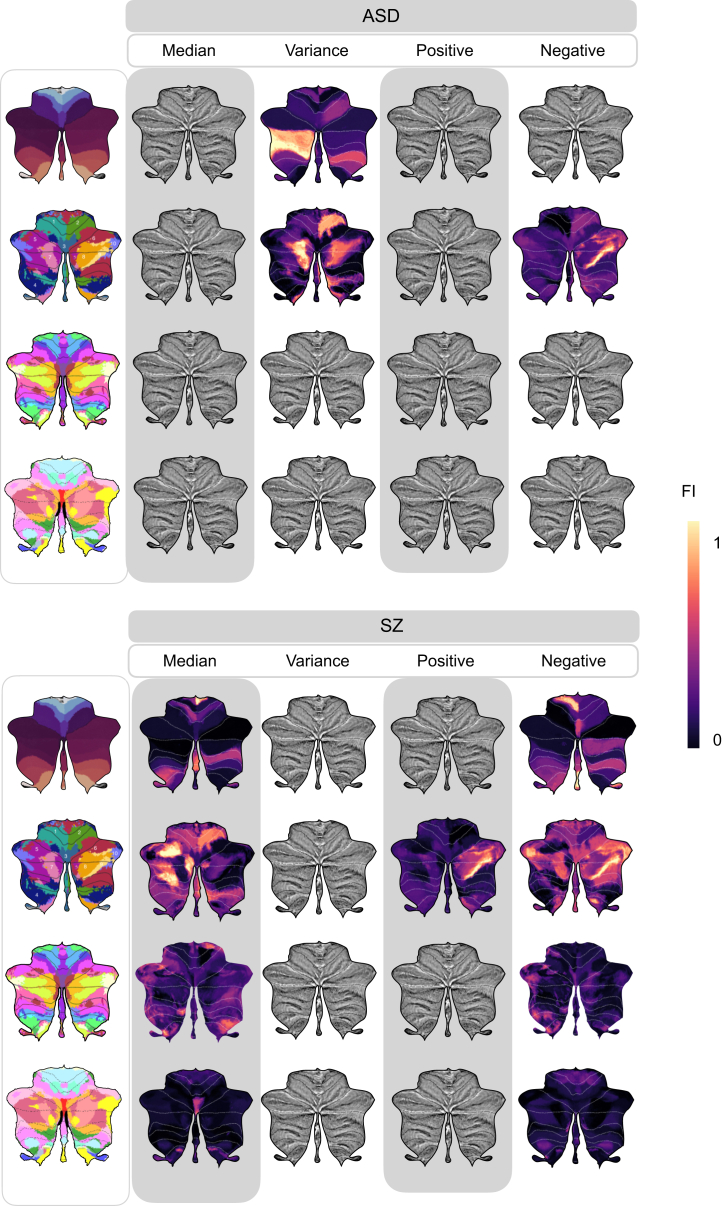
Different regions show distinct feature importance (FI) across atlases in autism spectrum disorder (ASD) and schizophrenia (SZ). The FI values derived from logistic regression reveal the contribution of each specific cerebellar region to predictive modeling relative to average prediction outcomes. FI values accentuate distinct cerebellar regions with unique predictive capabilities as identified in lobules, anatomical, task-based, hierarchical, and resting-state atlases through voxelwise analysis. Features that remained significant after adjustments for multiple comparisons of the area under the receiver operating characteristic curve are shown.

Figure 3 presents the standardized feature importance weights in an LR model used to analyze SZ and ASD (see Figure S2 for all feature importance). For each analysis of lobules or atlases, we report the 2 highest-ranking features. In SZ, significant negative deviation percentages were found in the vermis IX and left IV regions in the anatomical atlas. In task-based functional areas, regions associated with verbal fluency, word comprehension, and mental arithmetic (region 9) and autobiographical recall, visual letter recognition, and interference resolution (region 10) were notable. The 2 highest-ranking feature importance in the hierarchical atlas were found in the left S1 (social-linguistic-spatial) and left D1 (demand) regions. From the resting-state atlas, limbic A (region 10) and somatomotor A (region 3) emerged as important. For the median in SZ, the anatomical regions right I–III and vermis VIII were highlighted. Using task-based atlases, the top predictive regions were functionally linked to divided attention (region 5) and right-hand movement (region 2). In the hierarchical atlas, the median values were observed in the same regions as the percentage of extreme negative deviations. Predictive models using an atlas based on resting-state atlas highlighted visual B (region 2) and limbic A (region 10).

In ASD, predictive models based on regional variance revealed the 2 highest-ranking feature importance from posterior cerebellar regions of left VIIB and left crus II in the anatomical atlas, while models based on lobular volume features point to right VI and left crus II. Using the task-based functional atlas, the most predictive regions were functionally linked to narrative, emotion, and language processing (region 7) and right-hand movement, motor planning, and divided attention (region 2). Complementary insights and detailed rankings of feature importance are available in Tables S5 to S9.

## Discussion

In this study, we aimed to test the predictive power of deviations from normal cerebellar anatomy with respect to classifying mental and neurological disorders and yielded 2 main findings. First, we demonstrated that cerebellar features offered moderate power for prediction of ASD and SZ but did not reliably distinguish reference samples from patients with BD, MCI, or AD. Second, feature importance analyses showed that both anterior and posterior regions of the cerebellum were dominant features of ASD and SZ.

Our study reveals that features derived from lobular and voxelwise normative models possess moderate predictive capabilities in ASD and SZ. This is consistent with our previous study, which revealed small to medium case-control differences in normative cerebellar anatomy for both ASD and SZ (15). On the other hand, as functional topography does not consistently adhere to anatomical boundaries in the cerebellum, we also examined task-based, hierarchical, and resting-state atlases in voxelwise normative space. As a result, no single atlas consistently emerged as superior to the others. However, we believe that using various atlases aids in the interpretation of our findings.

A comprehensive feature importance analysis for predicting SZ highlighted significant contributions from regions associated with both motor (56,57) and cognitive (20) functions. Within the anatomical atlas superimposed on the voxelwise cerebellar maps, vermis IX and left IV exhibited the 2 highest-ranking feature importance for percentage of extreme negative deviations, while right I–III and vermis VIII were most prominent in median values. Median features, which reflect the central tendency across *z* scores within one region, largely overlapped with those identified in the analysis of extreme negative deviations, which measure divergence from normative values. Notably, vermis IX has consistently been reported to exhibit reductions in individuals with SZ, underscoring its potential role in the disorder’s pathology (58, 59, 60, 61). These findings are consistent with previous research suggesting that the limbic vermis plays a crucial role in emotional processing, facial expression recognition (62, 63, 64), and mentalizing—the ability to understand others’ mental states (22,64, 65, 66). Recent studies (67) have also highlighted cerebellar hypoplasia, primarily affecting the posterior vermis, in individuals with SZ or undifferentiated psychosis. Additionally, the anterior cerebellum, including lobules I–IV, has established connections with the primary motor cortex, which is critical for motor function (68). This connection may explain the motor dysfunctions in SZ, such as impairments in eye-blink conditioning, timing, postural control, and motor learning, which are associated with reduced volumes in anterior cerebellar regions and the vermis (69, 70, 71). The involvement of both motor and cognitive regions underscores the disorder’s complexity, providing deeper insights into its diverse facets.

When the voxelwise cerebellar maps were mapped onto a task-based atlas for extreme negative deviations (23), regions linked to verbal fluency and autobiographical recall were identified, while divided attention and right-hand movement were associated with median deviations in SZ. In a hierarchical atlas, models of extreme negative deviation and median feature importance were predominantly highlighted in the left S1 (social-linguistic-spatial) and left D1 (demand) regions, which emphasize linguistic processing and verb generation. A recent study (18) linked language difficulties in early psychosis to alterations in the right posterolateral cerebellar region, an area involved in verb generation and cognitive functions such as attention and memory (23). This suggests that individuals with early psychosis may struggle with verbal expression and thought control, impairing perceptual processing. These regions overlap with those previously associated with speech perception and production (72).

Mapped to the resting-state atlas, the most significant features included limbic A and somatomotor A in terms of extreme negative deviations, while visual B and limbic A were notable for median values. The limbic network is involved in emotion processing, memory, and behavior, while the somatomotor network governs motor processing and execution, and the visual network processes visual information. SZ is often accompanied by oculomotor abnormalities, affecting eye movement control in response to visual stimuli and anticipated actions (73). Studies of cerebro-cerebellar connectivity have reported increased connectivity within somatomotor, sensorimotor, and default mode networks but decreased connectivity in higher-order networks, including attention, salience, and executive control regions (74,75). While direct comparisons between functional connectivity studies and structural analyses may be challenging due to methodological differences, these findings remain relevant.

Previous studies have reported inconsistent and hard-to-replicate findings on the relationship between cerebellar volume and ASD (15,76). Interestingly, ASD was best predicted by the variance of *z* scores within individual parcellations of the cerebellum, indicating greater voxel-level variability in different regions. Feature importance analyses in ASD highlighted left VIIB and left crus II in the anatomical atlas, as well as narrative, emotion, and language processing regions and right-hand movement in the task-based atlas. Crus I–II and lobule VIIB are densely connected to prefrontal and parietal cortices through cerebello-thalamo-cortico-pontine circuits (77) that are critical for higher-level processes. Its substantial heterogeneity, ranging from high-functioning individuals to individuals who require significant support, presents challenges in identifying a common neurobiological basis (78).

Prior research has consistently demonstrated strong classification of dementia using whole-brain imaging data, with AUROC values ranging from 0.904 to 0.920 (3). Thus, the absence of significant predictive models for BD, MCI, and AD was unexpected, although it is worth noting that the effects of MCI and AD were relatively small in our previous study as well (15). While null findings should be interpreted with caution, the lack of effects in a moderately large sample of baseline patients with AD suggests that the cerebellum may be relatively spared (79,80). However, both typical aging and AD are associated with gray matter loss in the cerebellum, particularly in crus I–II and lobule VI. In typical aging, this decline occurs bilaterally, whereas in AD, it is more pronounced in the right hemisphere (81). Given the growing research on cerebellar aging (82), additional studies are needed to determine how MCI and AD pathology contribute to cerebellar atrophy and to what extent the cerebellum remains relatively preserved. Similarly, research on BD and its cerebellar involvement is hindered by inconsistent findings (83), underscoring the need for more comprehensive investigations.

There are limitations to consider in our study. First, harmonizing behavioral, cognitive, genetic, phenotypic, lifestyle, symptomatology, and medical history data across various datasets poses significant challenges, especially when aiming for a large sample size essential for assessing generalizability. The limited predictive power of the trained machine learning models (84) should be taken into consideration when interpreting the current findings. In multivariate models, feature importance must be assessed with regard to the overall model context. Collinearity among features can result in fluctuations in their rankings, necessitating cautious interpretation. For practicality, we have concentrated our discussion on the 2 features that emerged as highest in ranking. Next, accurately classifying complex clinical conditions is challenging due to the intrinsic heterogeneity of these conditions, which manifests as a wide array of symptoms and genetic variations. Some individuals may exhibit resilience due to genetic or lifestyle factors, which can complicate accurate predictions (85). The key challenge in normative modeling lies in interpreting deviations at the individual level. The central question is whether these deviations are biologically significant or merely artifacts resulting from signal dropout during imaging. Therefore, individual-level interpretation is still an area under active development. Furthermore, the existence of subgroups within heterogeneous conditions, such as ASD, complicates the interpretation of performance metrics of prediction models. Neurodevelopmental changes raise concerns about the appropriateness of applying adult template space and atlases to younger children and adolescents (86). Including other key brain regions and utilizing a multimodal approach that integrates different types of brain imaging data may improve predictions (87,88). Future studies should compare cerebellar and whole-brain models to clarify the cerebellum’s unique contribution within the broader neural context. Given the large sample size, we manually inspected about 5% of the scans to maintain quality control. Nevertheless, we acknowledge the limitations in accurately depicting different atlases with varying border including lobular segmentation. The cerebellum’s distinct position within the skull and its intricate folding pattern also present challenges in obtaining precise MRI data. Because we incorporated a large number of samples, the normative models are not affected by occasional outliers that might have been missed in our extensive quality control procedures, which additionally strengthens our confidence in the robustness of the reported findings. Finally, an AUROC value in the range of 0.7 to 0.8 can be deemed acceptable for certain clinical applications (89), indicating fair discrimination that includes the range of our model. However, for many clinical scenarios, this may not suffice, as values from 0.8 to 0.9 are generally regarded as appropriate (90). Future research efforts should aim to address these limitations and further enhance our understanding of predictive models.

### Conclusions

In this study, we tested the value of cerebellar-derived features for predictions of 5 mental and neurological conditions. The analysis revealed moderate prediction performance for ASD and SZ, with strongest contributions across cerebellar regions aided by 4 cerebellar atlases that enhanced the interpretability of the findings.

## Supplementary Material

Supplemental Methods, Figures S1–S4, and Tables S1–S9

Key Resources Table
