# Supplementary material for: Predicting Mental and Neurological Illnesses Based on Cerebellar Normative Features
Source: Biol Psychiatry Glob Open Sci. 2025 May 28;5(5):100541. doi: 10.1016/j.bpsgos.2025.100541 (PMC12268537; doi:10.1016/j.bpsgos.2025.100541)
Supplement: Supplemental Methods, Figures S1–S4, and Tables S1–S9 [file mmc1.pdf]

SUPPLEMENTARY INFORMATION

Predicting Mental and Neurological Illnesses Based on Cerebellar Normative Features

Kim *et al.*

Table of Contents

Supplementary Methods

List of Supplementary Figures

1. Labelled cerebellar atlases used in the study
2. Feature importance of all atlases
3. Between model comparison in anatomical atlas
4. Comparison with Random Forest in anatomical atlas

List of Supplementary Tables

1. Sources of the studies used in the study
2. Full sample description and demographics
3. Descriptive statistics of cerebellar lobular z-scores
4. AUROC values of each clinical cohorts
5. Feature Importance of ASD
6. Feature Importance of BD
7. Feature Importance of SZ
8. Feature Importance of MCI
9. Feature Importance of AD

Supplementary Methods

Statistics & Reproducibility

The quality control of this study is identical the procesude from Kim and colleagues(1) utilizing the full sample of the cerebellar normative model. We performed quality control by running the ENIGMA Cerebellum Volumetric Pipeline QC Scripts of ACAPULCO(2) with Singularity(3). Given the extensive volume of brain scans available, amounting to thousands, it was not feasible to manually inspect each individual scan. However, approximately 5% of these scans were selected and examined manually to ensure quality and consistency within the dataset. The "QC_Images.html" file displays visual representations of the segmented images in coronal, sagittal, and transverse sections, which allows for a thorough inspection of the segmentation quality. The QC pipeline delivers both quantitative and visual information aids pertaining to the volumetric aspects of the cerebellum's segmented regions including volume, outliers, and box plots of the outliers. By integrating quality control pipeline into our analysis, it enabled us to detect and rectify any mis-segmentations or statistical outliers, thereby enhancing the dependability and precision of our findings. We further excluded participants whose scans revealed outliers in at least two regions, as well as when a scanning site contributed data from fewer than five participants.

Atlases

The cerebellum's complex structure suggests that its various subregions have distinct roles in different cognitive and motor tasks. Functional subregions within the cerebellum do not correspond neatly with its lobular architecture. As shown in Multi-Domain Task-Battery (MDTB) map(4), specific areas are linked to discrete functions: regions 1 and 2 with hand movements, 3 with visual memory, 4 with attention, 5 and 6 with working memory, 7 and 8

with narrative comprehension, 8 and 9 with language functions, and region 10 with autobiographical recall. These functional delineations show that the organization of cerebellar functions transcends classic anatomical divisions, with functional domains intersecting various lobules.

The recently updated hierarchical atlas by Nettekoven (5) incorporates data from seven extensive datasets, offering a more comprehensive perspective compared to previous atlases that primarily focused on single task-based or resting-state fMRI. This advanced atlas provides both asymmetrical and symmetrical versions into 4 domains, 32 regions, 68 functional subregions, and 128 spatial subregion parcellations, which are essential for comprehending the intricate structure of the cerebellum. The four domains consist of Motor (M), Action (A), Demand (D) and Social-linguistic-spatial (S). The Motor (M) domains encompass M1, which is associated with saccades and visual stimuli, M2, which responds to tongue movements, M3, which facilitates the movement of the ipsilateral hand, and M4, which involves the movement of the lower body. The Action (A) domains comprise A1, linked to spatial stimulation, A2, related to action observation, and A3, connected with motor imagery. The Demand (D) domains include D1, pertinent to spatial tasks, D2, concerned with backward recall, D3, associated with working memory load, and D4, involved in the n-back test. The Social-Linguistic-Spatial (S) domains consist of S1, which is engaged in linguistic processing, such as verb generation, S2, which facilitates social processing, specifically theory of mind, while S3 and S4 are significantly active during rest, akin to the default network B's theory of mind. Lastly, S4 and S5 are involved in imagination and self-projection during spatial and motor imagery tasks; notably, S5 is activated by action observation.

Buckner et al. (6,7) highlighted the intricate patterns of connectivity between the cerebellum and the cerebral cortex during the resting state. Resting state networks demonstrate shared functional activation despite the absence of explicit tasks. Moereover, cerebellum's

participation in continuous functional interactions with the cerebral cortex are crucial for overall brain functionality. The studies have unraveled a comprehensive topographic organization of the cerebellum, with the cerebellar lobules showing specific connectivity patterns with various cortical areas, underpinning the cerebellum's involvement in a spectrum of both motor and non-motor functions. Lobule VIII and the anterior lobe of the cerebellum are connected with cortical and premotor areas that are parts of networks 3, 4, and 7, which are associated with somatomotor and attentional functions. Conversely, lobules VII and IX of the cerebellum are linked with prefrontal and parietal association areas, engaging in networks 8, 12, 13, 14, 16, and 17. The regions for the 17 networks are N1: Visual A, N2: Visual B; Network 3: Somatomotor A; N4: Somatomotor B; N5: Dorsal Attention A; N6: Dorsal Attention B; N7: Salience/ Ventral Attention A; N8: Salience/ Ventral Attention B; N9: Limbic B; N10: Limbic A; N11: Control A; N12: Control B; N13: Control C; N14: Default A; N15: Default B; N16: Default C; N17: Temporal Parietal.

Supplementary Figures

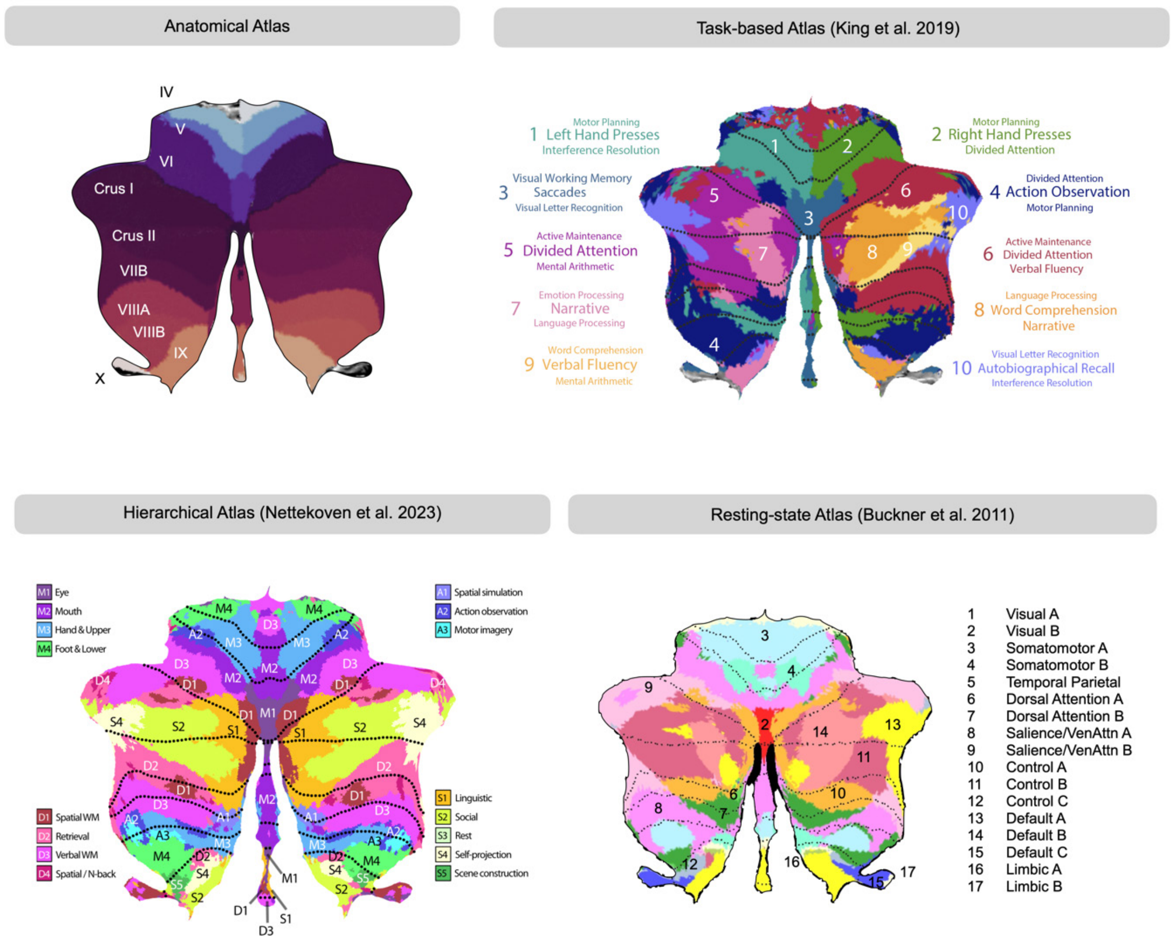

Supplementary Figure 1. Labelled Cerebellar Atlases used in the study. In this study, we utilized a variety of cerebellar atlases, including anatomical, task-based, hierarchical, and resting-state atlases. For more detailed information, please refer to the Supplementary Methods section on atlases.

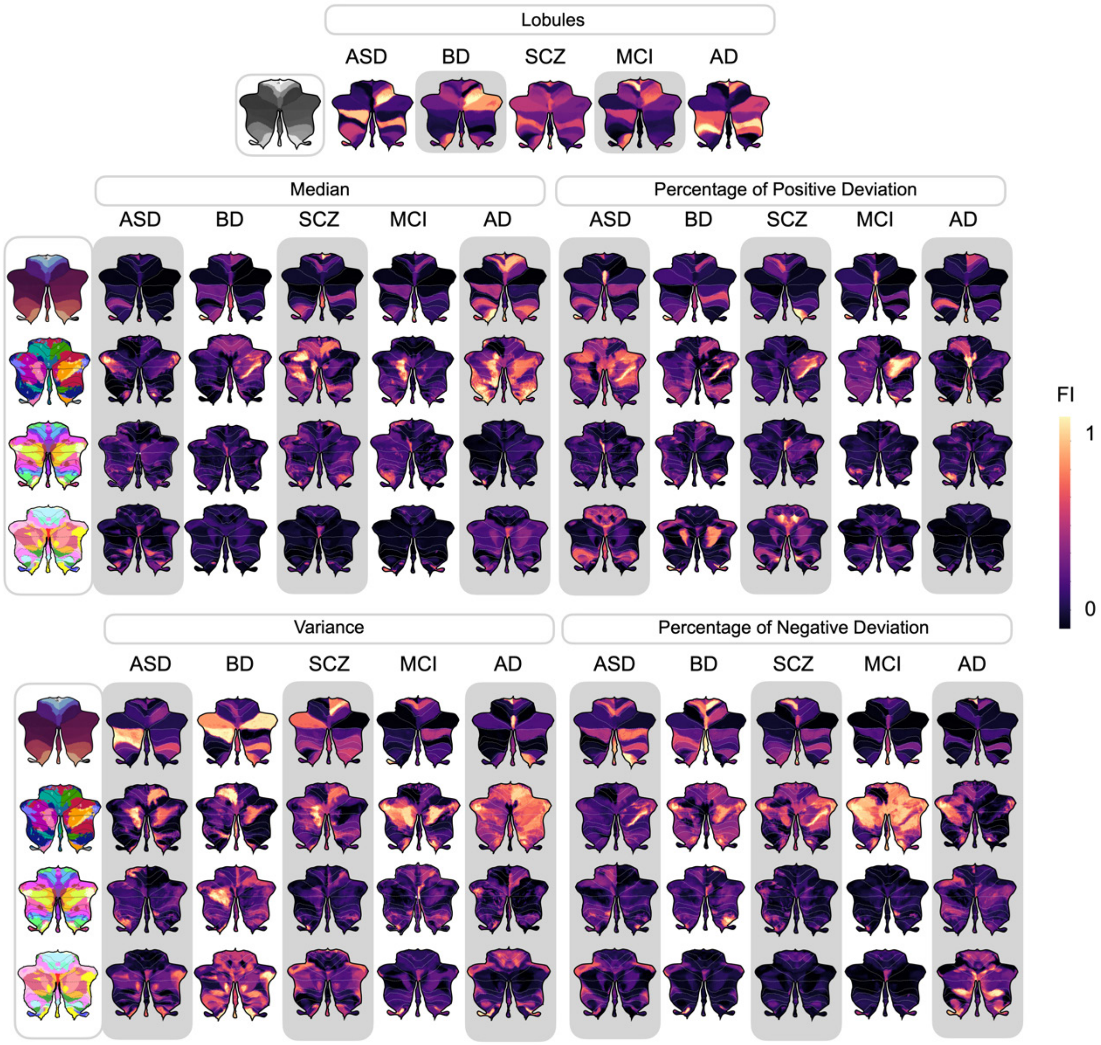

Supplementary Figure 2. Feature importance of all disorders and atlases shown. Both features that remained significant and non-significant after adjustments for multiple comparisons of AUROC are shown.

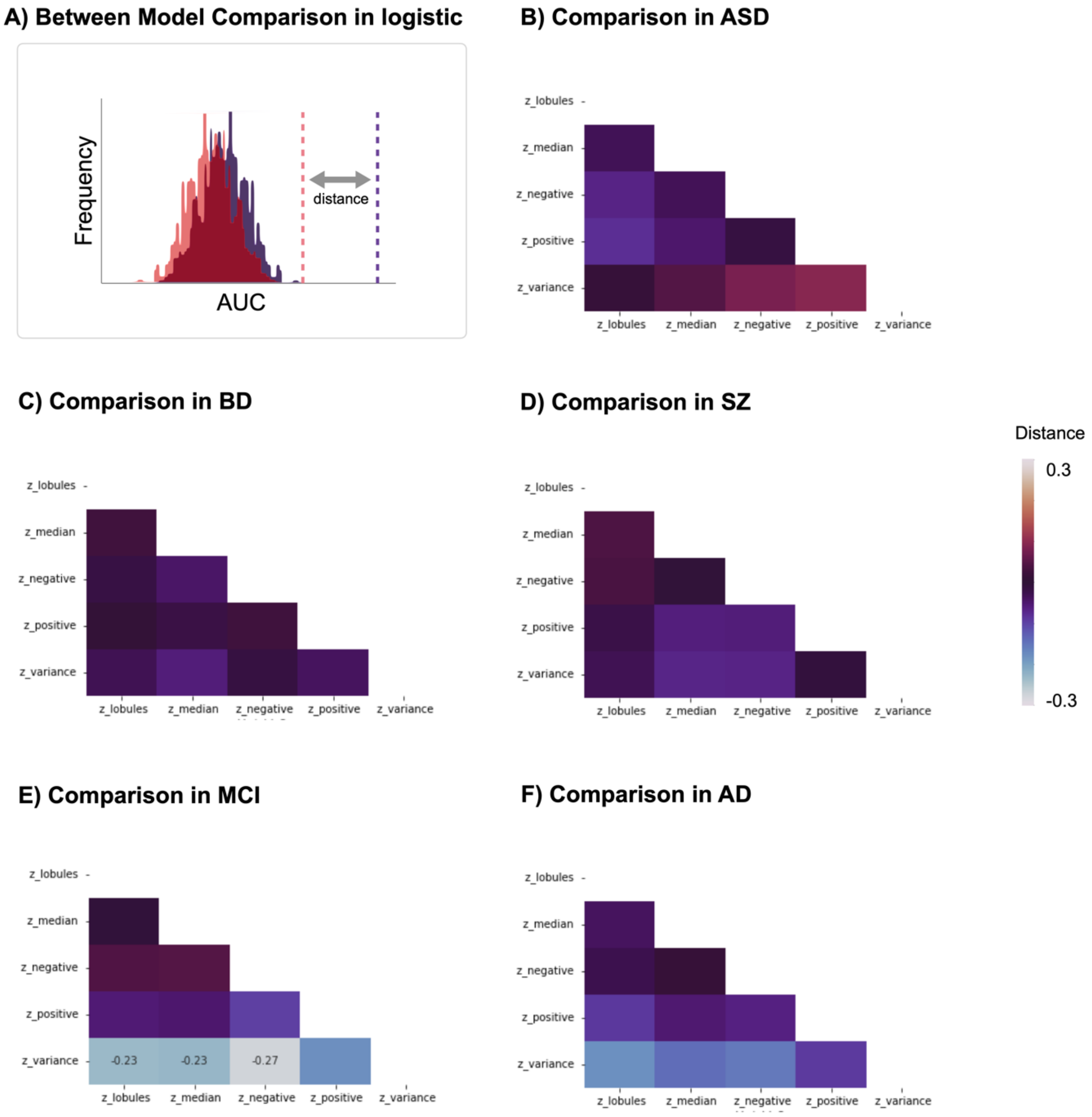

Supplementary Figure 3. Comparisons between models within the anatomical atlas generally yield similar performance. (A) The figure illustrates the comparative distance in performance between pairs of models that have withstood multiple comparison tests for each disease across anatomical atlas. (B) This suggests that while most models are equally predictive, the variance model is an exception in the MCI cohort.

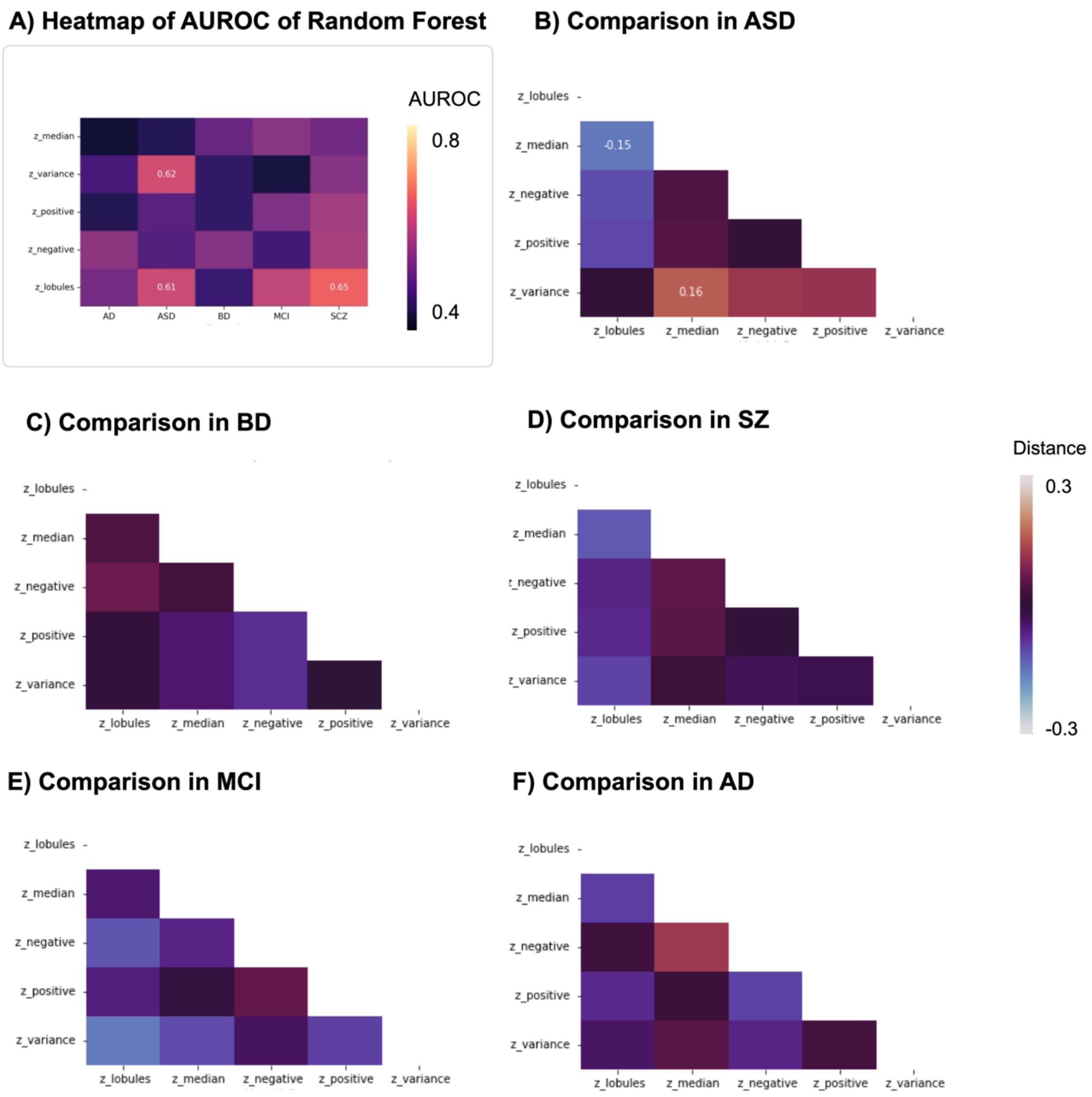

Supplementary Figure 4. Random Forest achieves a performance comparable to that of Logistic Regression in anatomical atlas (A) The heatmap of the AUROC for the Random Forest model displaying results akin to linear regression. (B-F) Also, comparisons between different models using Random Forest also indicating no significant differences except in ASD.

Supplementary Tables

Supplementary Table 1. Sources of the studies used in the study

Datasets	Sources	Comments	References
Autism Brain Imaging Dataset Exchange (ABIDE-I)	http://fcon_1000.projects.nitrc.org/	Primary support for the work by Adriana Di Martino was provided by the NIMH (K23MH087770) and the Leon Levy Foundation. Primary support for the work by Michael P. Milham and the INDI team was provided by gifts from Joseph P. Healy and the Stavros Niarchos Foundation to the Child Mind Institute, as well as by an NIMH award to MPM (R03MH096321).	(8)
Autism Brain Imaging Dataset Exchange II (ABIDE-II)	http://fcon_1000.projects.nitrc.org/	Primary support for the work by Adriana Di Martino and her team was provided by the National Institute of Mental Health (NIMH 5R21MH107045). Primary support for the work by Michael P. Milham and his team provided by the National Institute of Mental Health (NIMH 5R21MH107045); Nathan S. Kline Institute of Psychiatric Research). Additional Support was provided by gifts from Joseph P. Healey, Phyllis Green and Randolph Cowen to the Child Mind Institute.	(9)
Alzheimer's Disease Neuroimaging Initiative (ADNI)	http://adni.loni.usc.edu/	The ADNI was launched in 2003 as a public-private partnership, led by Principal Investigator Michael W. Weiner, MD. ADNI consists of 4 waves, the later is still ongoing (ADNI 3). A complete listing of ADNI investigators can be found at http://adni.loni.usc.edu/wpcontent/uploads/how_to_apply/ADNI_Acknowledgement_List.pdf. Data collection and sharing for this project was funded by the Alzheimer's Disease Neuroimaging Initiative (ADNI) (National Institutes of Health Grant U01 AG024904) and DOD ADNI (Department of Defense award number W81XWH-12-2-0012). ADNI is funded by the National Institute on Aging, the National Institute of Biomedical Imaging and Bioengineering, and through generous contributions from the following: AbbVie, Alzheimer's Association; Alzheimer's Drug Discovery Foundation; Araclon Biotech; BioClinica, Inc.; Biogen; Bristol-Myers Squibb Company; CereSpir, Inc.; Cogstate; Eisai Inc.; Elan Pharmaceuticals, Inc.; Eli Lilly and Company; EuroImmun; F. Hoffmann-La Roche Ltd and its affiliated company Genentech, Inc.; Fujirebio; GE Healthcare; IXICO Ltd.;Janssen Alzheimer Immunotherapy Research & Development, LLC.; Johnson & Johnson Pharmaceutical Research & Development LLC.; Lumosity; Lundbeck; Merck & Co., Inc.;Meso Scale Diagnostics, LLC.; NeuroRx Research; Neurotrack Technologies; Novartis Pharmaceuticals Corporation; Pfizer Inc.; Piramal Imaging; Servier; Takeda Pharmaceutical Company; and Transition Therapeutics. The Canadian Institutes of Health Research is providing funds to support ADNI clinical sites in Canada. Private sector contributions are facilitated by the Foundation for the National Institutes of Health (www.fnih.org). The grantee organization is the Northern California Institute for Research and Education, and the study is coordinated by the Alzheimer's Therapeutic Research Institute at the University of Southern California. ADNI data are disseminated by the Laboratory for Neuro Imaging at the University of Southern California.	
The Australian Imaging, Biomarkers and Lifestyle Flagship Study (AIBL)	https://aibl.csiro.au/	Australian Imaging Biomarkers and Lifestyle flagship study of ageing (AIBL) was funded by the Commonwealth Scientific and Industrial Research Organisation (CSIRO), which was made available at the ADNI databas (http://www.loni.usc.edu/ADNI). The AIBL researchers contributed data but did not participate in analysis or writing of this report. AIBL researchers are listed at http://www.aibl.csiro.au. Correspondence should be addressed to Christopher Rowe (email: christopher.rowe@austin.org.au).	(10)
Norwegian Dementia Genetics Network (DEMGEN)	Authors	Supported by the Norwegian National Advisory Unit on Aging and Health	(11,12)

StrokeMRI (TOP)	Authors	Supported by the Research Council of Norway (249795, 248238), the South-Eastern Norway Regional Health Authority (2014097, 2015044, 2015073, 2016083), and the Norwegian ExtraFoundation for Health and Rehabilitation (2015/FO5146)	(13)
Thematically Organized Psychosis (TOP)	Authors	Supported by several grants from the Research Council of Norway, and the South-Eastern Norway Regional Health Authority	(14–16)

Supplementary Table 2. Full Sample description and demographics

		N (Participants)	N (Scanning-site)	Age (Mean, S.D.)	Sex (%F:%M)
Full	All	54102	132		
	Training set	27117	132	54.36 (20.31)	53:47
	Testing set	26985	132	54.52 (20.19)	53:47
Clinical	Testing set	1757	53	29.40 (20.84)	30:70
	Alzheimer's Disease (AD)	146	13	72.42 (7.65)	53:47
	Autism Spectrum Disorder (ASD)	900	37	16.20 (9.00)	14:86
	Bipolar Disorder (BD)	277	3	32.73 (11.67)	60:40
	Mild Cognitive Impairment (MCI)	122	3	67.25 (9.27)	42:58
	Schizophrenia (SZ)	312	3	29.58 (9.52)	33:67

Supplementary Table 3. Descriptive Statistics of Cerebellar Lobular Z-scores

a) ASD and matched HC

diagnosis	ROI	mean	median	std	min	max
ASD	Corpus.Medullare	-0.0403	-0.0296	0.9822	-3.0611	2.8145
HC	Corpus.Medullare	-0.0104	-0.0388	0.8972	-2.6006	3.4816
ASD	Left.Crus.I	-0.0546	-0.0128	1.0312	-4.1766	3.1066
HC	Left.Crus.I	-0.0068	0.0302	0.9549	-4.0041	3.1490
ASD	Left.Crus.II	-0.0707	0.0293	1.1953	-9.4457	3.0855
HC	Left.Crus.II	0.0887	0.0954	1.0062	-5.3437	3.1894
ASD	Left.I.III	-0.0384	-0.0069	0.9905	-3.2908	3.2800
HC	Left.I.III	-0.0158	0.0321	0.9874	-4.1883	2.7317
ASD	Left.IV	-0.0521	-0.0784	0.9856	-3.0846	2.5621
HC	Left.IV	-0.0599	-0.0052	0.9314	-3.2025	2.3664
ASD	Left.IX	-0.0219	0.0272	1.0938	-6.2542	2.8406
HC	Left.IX	0.0529	0.1242	0.9965	-6.6146	2.7487
ASD	Left.V	-0.1525	-0.2045	1.0497	-4.2118	2.7252
HC	Left.V	-0.0842	-0.1137	0.9467	-2.9856	2.8627
ASD	Left.VI	-0.0320	0.0030	1.0191	-3.2524	3.2096
HC	Left.VI	-0.0176	-0.0373	1.0163	-3.5608	3.6861
ASD	Left.VIIB	-0.1576	-0.0032	1.6350	-9.5741	5.5068
HC	Left.VIIB	0.0581	0.0546	1.1842	-6.5324	4.1523
ASD	Left.VIIIA	-0.1337	0.0060	1.6868	-11.1316	7.2602
HC	Left.VIIIA	0.1215	0.1655	1.2788	-7.4786	5.4215
ASD	Left.VIIIB	-0.1353	0.0152	1.4770	-8.2907	3.6063
HC	Left.VIIIB	0.0485	0.1129	1.1455	-6.9960	3.6655
ASD	Left.X	-0.0524	-0.0226	1.1584	-8.4123	3.4638
HC	Left.X	0.0888	0.1285	1.0473	-7.5222	2.7056
ASD	Right.Crus.I	-0.0710	-0.0206	1.0645	-4.4033	3.2278
HC	Right.Crus.I	0.0326	0.0269	0.9926	-3.9370	2.8300
ASD	Right.Crus.II	-0.1838	-0.0165	1.3842	-9.1836	3.1906
HC	Right.Crus.II	0.0822	0.1381	1.0412	-5.2260	2.7797
ASD	Right.I.III	-0.0276	0.0182	0.9853	-2.8482	2.6548
HC	Right.I.III	0.0092	-0.0355	0.9886	-3.0410	3.2773
ASD	Right.IX	0.0356	0.0848	1.0698	-4.0841	3.3241
HC	Right.IX	0.0638	0.0955	1.0251	-7.9976	3.4444
ASD	Right.V	-0.2058	-0.1823	1.0224	-3.5975	2.9828
HC	Right.V	-0.0567	-0.0662	0.9484	-3.6480	2.5879
ASD	Right.VI	0.0460	0.0693	0.9739	-3.1350	3.0078

HC	Right.VI	-0.0482	-0.0411	0.9992	-3.3329	3.3797
ASD	Right.VIIB	-0.0835	0.0938	1.4881	-8.3816	4.2649
HC	Right.VIIB	0.0166	0.0612	1.0832	-10.1520	3.6082
ASD	Right.VIIIA	0.0357	0.1352	1.3820	-7.7232	4.1528
HC	Right.VIIIA	0.0719	0.0829	1.1371	-4.5216	5.0993
ASD	Right.VIIIB	-0.1060	-0.0116	1.3460	-5.4186	4.1801
HC	Right.VIIIB	0.0149	0.0530	1.1047	-4.8769	3.6921
ASD	Right.X	-0.0439	-0.0183	1.0586	-5.8702	3.8757
HC	Right.X	0.0474	0.0397	1.0283	-5.8673	3.2078
ASD	Rigt.IV	-0.0477	-0.0553	0.9740	-3.0871	3.1556
HC	Rigt.IV	-0.0008	0.0156	0.9282	-2.7900	2.8005
ASD	Vermis.IX	-0.0692	0.0044	1.0587	-5.5047	3.1724
HC	Vermis.IX	-0.0065	0.0232	0.9684	-3.4268	2.9445
ASD	Vermis.VI	-0.0223	-0.0243	1.0076	-3.3503	3.1962
HC	Vermis.VI	-0.0138	0.0156	0.9555	-3.8668	2.5951
ASD	Vermis.VII	-0.0922	-0.0762	0.9345	-3.5774	2.5060
HC	Vermis.VII	-0.0228	-0.0239	0.9277	-2.8934	3.1408
ASD	Vermis.VIII	0.0061	0.0311	0.9964	-3.2315	3.1455
HC	Vermis.VIII	0.0753	0.0491	0.9733	-3.5291	3.3432
ASD	Vermis.X	-0.1380	-0.1204	1.1091	-6.3666	2.7782
HC	Vermis.X	-0.0045	0.0382	0.9878	-3.6529	2.9900
ASD	Total_Cerebel_Vol	-0.0931	-0.0130	1.1486	-4.6441	3.2124
HC	Total_Cerebel_Vol	0.0409	0.0755	0.9599	-4.0446	2.6886

b) BD and matched HC

diagnosis	ROI	mean	median	std	min	max
BD	Corpus.Medullare	0.0711	0.0993	0.8514	-2.7175	2.3776
HC	Corpus.Medullare	0.0605	0.0949	0.9000	-2.5388	2.0618
BD	Left.Crus.I	-0.1374	-0.0903	0.8593	-2.7164	1.6476
HC	Left.Crus.I	-0.0984	-0.1142	0.8744	-2.5861	2.6036
BD	Left.Crus.II	-0.1181	-0.1026	0.9250	-2.3294	2.4487
HC	Left.Crus.II	-0.0954	-0.1066	0.8865	-2.3577	2.4910
BD	Left.I.III	-0.0067	-0.0082	0.9378	-2.6966	2.0438
HC	Left.I.III	-0.0764	-0.0790	0.9315	-2.4726	2.3676
BD	Left.IV	-0.0943	-0.1019	0.9478	-3.2039	2.3573
HC	Left.IV	-0.1033	-0.1262	0.8753	-2.5778	2.1957
BD	Left.IX	-0.1216	-0.1748	1.0053	-2.8311	2.6862

HC	Left.IX	-0.0712	-0.0613	1.0152	-6.6146	2.4514
BD	Left.V	-0.0270	0.0026	1.0054	-2.9195	2.1150
HC	Left.V	-0.0320	-0.0696	0.9614	-2.9614	2.5534
BD	Left.VI	-0.0612	-0.0495	0.9111	-2.9306	1.9653
HC	Left.VI	-0.0383	-0.0720	0.9803	-2.7918	2.4343
BD	Left.VIIB	-0.0443	-0.1151	0.8766	-2.4582	2.1709
HC	Left.VIIB	-0.0220	0.0490	0.8910	-2.6268	2.6598
BD	Left.VIIIA	-0.0061	-0.0246	0.9561	-3.2873	2.2714
HC	Left.VIIIA	-0.0627	-0.0527	0.9267	-2.8703	2.4709
BD	Left.VIIIB	-0.0169	0.0111	0.9963	-3.2236	3.1887
HC	Left.VIIIB	-0.0146	0.0435	0.9162	-2.4902	2.3591
BD	Left.X	0.0274	0.0066	0.8655	-2.4441	2.1594
HC	Left.X	-0.0849	-0.1087	1.0263	-6.5559	2.4372
BD	Right.Crus.I	-0.0353	-0.0789	0.8404	-2.1541	2.0855
HC	Right.Crus.I	0.0089	-0.0319	0.9255	-2.7739	3.1191
BD	Right.Crus.II	-0.0876	-0.0595	0.8387	-2.4410	2.0902
HC	Right.Crus.II	-0.0940	-0.0752	0.8332	-3.1389	2.1725
BD	Right.I.III	0.0928	0.0764	0.9455	-2.1700	2.2938
HC	Right.I.III	-0.0175	-0.0385	0.9944	-2.6110	2.7858
BD	Right.IX	-0.0750	-0.0306	1.0159	-3.2393	2.4865
HC	Right.IX	-0.0477	-0.0222	1.0013	-5.6714	2.3382
BD	Right.V	-0.0836	0.0058	1.0173	-3.3561	2.1485
HC	Right.V	-0.0634	-0.1275	0.9574	-2.6063	2.7468
BD	Right.VI	-0.0732	-0.0873	0.9156	-2.1847	2.5198
HC	Right.VI	-0.0012	0.0623	0.9629	-3.0069	2.1514
BD	Right.VIIB	-0.0985	-0.1169	0.8968	-3.0688	2.6798
HC	Right.VIIB	-0.1320	-0.1934	0.8999	-2.2982	2.3693
BD	Right.VIIIA	-0.0505	-0.0185	0.8313	-2.5361	2.4096
HC	Right.VIIIA	-0.0850	-0.1222	0.9052	-3.4641	2.4905
BD	Right.VIIIB	-0.1069	-0.0455	0.9419	-3.3418	2.6653
HC	Right.VIIIB	-0.0875	-0.0973	0.8307	-3.2092	2.5296
BD	Right.X	0.0399	0.1046	0.8981	-2.2394	2.5908
HC	Right.X	-0.0841	-0.0306	1.0566	-4.5907	2.5299
BD	Rigt.IV	-0.0412	-0.0023	0.9718	-3.2828	2.6338
HC	Rigt.IV	-0.0156	0.0212	0.9622	-2.8885	2.3505
BD	Vermis.IX	-0.0514	-0.0204	1.0194	-2.9834	2.8517
HC	Vermis.IX	0.0017	0.0552	0.9574	-3.3903	2.6729
BD	Vermis.VI	-0.0872	-0.1088	0.9251	-2.3717	3.2292
HC	Vermis.VI	-0.0512	-0.0646	0.9145	-3.0327	2.1014
BD	Vermis.VII	-0.0061	-0.0326	0.8700	-2.3978	1.8309

HC	Vermis.VII	-0.0456	0.0358	0.8715	-2.8274	2.3099
BD	Vermis.VIII	-0.0270	-0.0916	1.0076	-2.7636	2.3856
HC	Vermis.VIII	-0.1285	-0.1221	0.9279	-3.2048	2.0538
BD	Vermis.X	0.0843	0.0790	0.9617	-2.2750	3.2610
HC	Vermis.X	0.0741	0.0381	1.0108	-2.6083	2.8719
BD	Total_Cerebel_Vol	-0.0929	-0.1139	0.8180	-2.5835	1.8526
HC	Total_Cerebel_Vol	-0.0766	0.0061	0.8457	-2.6498	2.2264

c) SZ and matched HC

diagnosis	ROI	mean	median	std	min	max
HC	Corpus.Medullare	0.1147	0.1209	0.8782	-2.0859	2.0618
SCZ	Corpus.Medullare	-0.0987	-0.0693	0.8746	-1.9996	2.3652
HC	Left.Crus.I	0.0225	-0.0125	0.8880	-2.5861	2.3660
SCZ	Left.Crus.I	-0.2741	-0.3595	0.9314	-3.4246	2.4703
HC	Left.Crus.II	-0.0634	-0.0939	0.8779	-2.1008	2.4910
SCZ	Left.Crus.II	-0.2831	-0.3113	0.9753	-2.8623	2.2403
HC	Left.I.III	-0.0797	-0.0786	0.9440	-3.0030	2.3676
SCZ	Left.I.III	-0.1233	-0.1164	0.9737	-2.9444	3.0219
HC	Left.IV	-0.1415	-0.1292	0.8904	-2.5778	2.1957
SCZ	Left.IV	-0.1560	-0.1516	1.0021	-2.3757	2.6486
HC	Left.IX	-0.0272	-0.0033	0.9738	-6.6146	2.4514
SCZ	Left.IX	-0.1820	-0.1138	0.9717	-3.0630	2.3014
HC	Left.V	-0.0505	-0.0296	0.9436	-2.9614	2.5534
SCZ	Left.V	-0.1905	-0.2185	0.9904	-3.4394	2.9663
HC	Left.VI	0.0380	-0.0051	0.9694	-2.7918	2.4343
SCZ	Left.VI	-0.2244	-0.1742	1.0325	-3.4582	2.7349
HC	Left.VIIB	0.0131	0.0520	0.8733	-2.6268	2.0840
SCZ	Left.VIIB	-0.3043	-0.2804	0.9219	-4.2056	2.3636
HC	Left.VIIIA	-0.0344	-0.0340	0.9621	-2.8703	2.6639
SCZ	Left.VIIIA	-0.1853	-0.2148	1.0646	-4.2544	2.3335
HC	Left.VIIIB	0.0293	0.1397	0.9565	-2.6168	2.8774
SCZ	Left.VIIIB	-0.1752	-0.1257	1.0148	-3.3211	2.1478
HC	Left.X	-0.0932	-0.0689	0.9611	-6.5559	2.2242
SCZ	Left.X	-0.1629	-0.2001	1.0333	-4.5792	2.8258
HC	Right.Crus.I	0.0983	0.1006	0.9003	-2.5635	3.1191
SCZ	Right.Crus.I	-0.1637	-0.1775	0.8993	-3.2779	2.0552
HC	Right.Crus.II	-0.0596	0.0012	0.8335	-3.1389	1.9063

SCZ	Right.Crus.II	-0.3442	-0.2340	0.9360	-3.2574	1.9028
HC	Right.I.III	0.0014	0.0196	0.9526	-2.6110	2.9099
SCZ	Right.I.III	-0.1280	-0.1533	0.9523	-3.2824	2.3926
HC	Right.IX	-0.0178	0.0655	1.0302	-6.0073	2.3382
SCZ	Right.IX	-0.1389	-0.1091	0.9753	-2.6584	2.2024
HC	Right.V	-0.0621	-0.1290	0.9531	-2.7517	2.7468
SCZ	Right.V	-0.2101	-0.1434	0.9684	-2.9757	2.0257
HC	Right.VI	0.0394	0.0856	0.9782	-3.0069	2.1514
SCZ	Right.VI	-0.1579	-0.0939	1.0280	-3.0845	2.9845
HC	Right.VIIB	-0.0602	-0.0922	0.8869	-2.2982	2.4745
SCZ	Right.VIIB	-0.3218	-0.3294	0.9506	-3.2564	2.2654
HC	Right.VIIIA	-0.0833	-0.0435	0.9281	-3.4641	2.4905
SCZ	Right.VIIIA	-0.1612	-0.1703	0.9480	-3.4494	2.7892
HC	Right.VIIIB	-0.0316	-0.0453	0.8842	-3.2092	2.5467
SCZ	Right.VIIIB	-0.1924	-0.1716	0.9727	-3.4342	2.4947
HC	Right.X	-0.0368	-0.0054	1.0561	-4.5907	2.8307
SCZ	Right.X	-0.1928	-0.2096	1.0432	-7.6055	2.3837
HC	Rigt.IV	0.0431	0.0495	0.9233	-2.8885	2.4559
SCZ	Rigt.IV	-0.1089	-0.0970	1.0300	-3.2512	2.6591
HC	Vermis.IX	0.0551	0.1601	0.9383	-3.3903	2.6729
SCZ	Vermis.IX	-0.3232	-0.2609	0.9465	-3.2285	2.5125
HC	Vermis.VI	0.0385	0.0261	0.9379	-3.0327	2.4823
SCZ	Vermis.VI	-0.1378	-0.0762	0.9763	-3.0848	3.1681
HC	Vermis.VII	0.0525	0.1237	0.8847	-2.4204	2.3099
SCZ	Vermis.VII	-0.1343	-0.1343	0.9900	-2.6927	2.3810
HC	Vermis.VIII	-0.0655	-0.1118	0.8929	-2.6638	2.0538
SCZ	Vermis.VIII	-0.2365	-0.2234	0.9772	-3.5570	2.7764
HC	Vermis.X	0.1020	0.1508	0.9791	-2.7757	2.8719
SCZ	Vermis.X	-0.2204	-0.1289	0.9604	-2.8839	2.4450
HC	Total_Cerebel_Vol	0.0093	0.0681	0.8327	-2.6498	2.0225
SCZ	Total_Cerebel_Vol	-0.3371	-0.3268	0.9202	-2.9085	2.2057

d) MCI and matched HC

diagnosis	ROI	mean	median	std	min	max
HC	Corpus.Medullare	0.1576	0.1392	1.0192	-2.3817	3.4816
MCI	Corpus.Medullare	-0.1716	-0.1449	1.0872	-2.5767	3.5562
HC	Left.Crus.I	0.0279	0.1492	0.9721	-2.2145	2.5293

MCI	Left.Crus.I	-0.1687	-0.1209	0.9151	-2.4278	1.7602
HC	Left.Crus.II	0.2352	0.1276	0.9685	-2.4685	2.6203
MCI	Left.Crus.II	-0.0480	-0.0619	0.8843	-2.3741	2.8549
HC	Left.I.III	0.1670	0.1767	0.9558	-1.9443	2.7317
MCI	Left.I.III	0.1309	0.1865	0.9872	-2.7698	2.1319
HC	Left.IV	-0.1795	-0.2508	0.9468	-2.0289	2.2617
MCI	Left.IV	-0.0828	-0.1798	1.0874	-3.0963	2.6734
HC	Left.IX	0.0528	-0.0041	1.0001	-2.5149	2.2153
MCI	Left.IX	0.0826	0.3222	0.9627	-2.7724	2.0680
HC	Left.V	-0.0496	-0.1919	0.9179	-2.0055	2.7499
MCI	Left.V	-0.2117	-0.0605	1.1626	-4.7244	2.4564
HC	Left.VI	0.0665	-0.0401	1.0778	-3.5608	2.5539
MCI	Left.VI	-0.1397	-0.0654	0.9814	-4.2660	2.3474
HC	Left.VIIB	0.0604	-0.0510	0.8845	-2.5036	2.4872
MCI	Left.VIIB	-0.2560	-0.1560	0.9756	-3.8763	2.6191
HC	Left.VIIIA	0.2518	0.2251	0.9949	-2.4787	2.6357
MCI	Left.VIIIA	0.1851	-0.0130	0.9557	-1.8018	2.6199
HC	Left.VIIIB	0.0147	0.0510	1.0582	-2.8545	2.1230
MCI	Left.VIIIB	-0.1088	0.1779	1.1858	-3.8267	2.1768
HC	Left.X	0.2141	0.3932	1.1005	-4.1671	2.4263
MCI	Left.X	0.0414	0.0599	0.9085	-2.9567	2.0034
HC	Right.Crus.I	-0.0197	-0.0351	1.0142	-2.3073	2.3057
MCI	Right.Crus.I	-0.2709	-0.2590	1.0859	-3.5784	2.2471
HC	Right.Crus.II	0.2580	0.3129	0.8374	-1.8874	2.5997
MCI	Right.Crus.II	-0.0522	0.0241	0.9224	-2.4605	1.8723
HC	Right.I.III	0.0598	0.0469	1.0688	-3.0410	3.2773
MCI	Right.I.III	0.0520	0.0877	1.0420	-2.3137	2.8268
HC	Right.IX	0.0491	0.0068	0.9733	-3.6034	2.0573
MCI	Right.IX	0.0356	0.0363	0.9508	-2.9090	2.3385
HC	Right.V	-0.3048	-0.3731	0.9786	-3.6480	2.1545
MCI	Right.V	-0.5339	-0.5277	1.1258	-4.9795	3.0747
HC	Right.VI	0.0720	0.0258	1.0263	-2.6692	2.2513
MCI	Right.VI	0.0297	0.0064	0.8857	-2.6301	2.3052
HC	Right.VIIB	0.2973	0.3519	0.8674	-2.1946	2.4171
MCI	Right.VIIB	0.0559	0.0665	0.8811	-3.1467	2.5404
HC	Right.VIIIA	0.3037	0.2873	0.8949	-2.2810	2.6845
MCI	Right.VIIIA	0.2936	0.2869	0.9163	-2.1426	2.7276
HC	Right.VIIIB	-0.1360	-0.1479	0.8803	-2.7500	2.0806
MCI	Right.VIIIB	-0.2530	-0.1997	1.1417	-3.4191	1.7984
HC	Right.X	0.2942	0.1876	1.0180	-2.7162	3.2078

MCI	Right.X	0.0596	0.0384	1.0671	-2.7236	2.9148
HC	Rigt.IV	0.1050	0.2148	0.9402	-2.3243	1.9620
MCI	Rigt.IV	-0.0668	-0.1862	0.9578	-3.3918	2.1335
HC	Vermis.IX	0.0206	0.0556	0.8454	-2.0211	2.6290
MCI	Vermis.IX	-0.0511	-0.0803	1.0942	-2.9308	2.1964
HC	Vermis.VI	-0.0831	0.0414	1.0575	-3.1575	2.3979
MCI	Vermis.VI	0.0044	-0.0212	0.9857	-2.3807	2.1841
HC	Vermis.VII	0.0475	0.1542	0.9049	-1.9439	2.6345
MCI	Vermis.VII	0.0270	0.0263	0.9789	-2.9532	2.4114
HC	Vermis.VIII	0.2396	0.1544	0.9440	-2.0879	2.6036
MCI	Vermis.VIII	0.1680	0.2454	1.0629	-2.2614	2.7688
HC	Vermis.X	0.0959	0.0905	0.9506	-2.6749	2.3445
MCI	Vermis.X	-0.0455	-0.0070	1.0296	-2.8957	2.1109
HC	Total_Cerebel_Vol	0.1647	0.1715	0.9164	-1.9001	2.5479
MCI	Total_Cerebel_Vol	-0.1190	-0.0272	0.9677	-3.0541	2.0295

e) AD and matched HC

diagnosis	ROI	mean	median	std	min	max
AD	Corpus.Medullare	-0.0648	-0.0094	0.9139	-2.4865	2.7167
HC	Corpus.Medullare	0.1386	0.1008	0.9508	-1.7286	3.4816
AD	Left.Crus.I	-0.3022	-0.2674	0.9551	-3.2408	1.9831
HC	Left.Crus.I	0.1492	0.1844	0.9439	-2.1947	2.5293
AD	Left.Crus.II	0.0039	0.0077	0.8637	-2.6261	2.0469
HC	Left.Crus.II	0.2303	0.1622	0.9498	-2.3021	2.6203
AD	Left.I.III	0.0674	0.0711	0.9942	-3.1118	2.3602
HC	Left.I.III	0.0071	0.0569	1.0193	-2.6855	2.7317
AD	Left.IV	-0.3002	-0.2634	0.8505	-2.9315	2.2070
HC	Left.IV	-0.0865	-0.1427	0.9726	-2.1798	2.3664
AD	Left.IX	0.0350	0.1420	1.2245	-8.5163	2.6749
HC	Left.IX	0.1442	0.1288	1.0337	-2.5149	2.7781
AD	Left.V	-0.1497	-0.1142	1.0461	-3.8841	2.7996
HC	Left.V	0.0431	0.0327	0.9088	-2.0055	2.7499
AD	Left.VI	-0.0892	-0.0407	0.9685	-2.7990	2.5635
HC	Left.VI	0.0829	-0.0088	1.0954	-3.5608	3.6861
AD	Left.VIIB	-0.2247	-0.2396	0.9747	-4.0008	1.8254
HC	Left.VIIB	0.0997	0.1174	0.8834	-2.5036	2.4872

AD	Left.VIIIA	0.2387	0.2383	0.8909	-2.4576	2.6682
HC	Left.VIIIA	0.1253	0.1179	0.9491	-2.2620	2.6357
AD	Left.VIIIB	0.0120	0.0459	0.9752	-2.8171	2.9365
HC	Left.VIIIB	0.0069	0.0155	1.0363	-2.8545	2.1230
AD	Left.X	0.3745	0.3912	0.9370	-2.6584	2.5822
HC	Left.X	0.2585	0.2943	1.0485	-4.1671	2.6816
AD	Right.Crus.I	-0.3531	-0.4474	1.0324	-2.8457	1.7404
HC	Right.Crus.I	0.1211	0.0807	0.9997	-2.4373	2.3057
AD	Right.Crus.II	0.0557	0.0780	0.8246	-2.0179	1.8554
HC	Right.Crus.II	0.2240	0.1790	0.8071	-1.8874	2.5997
AD	Right.I.III	-0.1834	-0.1774	0.9961	-3.0834	2.0349
HC	Right.I.III	-0.0783	-0.1124	1.0846	-3.0520	3.2773
AD	Right.IX	0.0402	0.0595	1.0180	-3.4313	2.7206
HC	Right.IX	0.1752	0.0675	1.0395	-3.6034	3.0812
AD	Right.V	-0.4781	-0.4118	1.1154	-3.4913	2.5101
HC	Right.V	-0.2316	-0.3041	1.0406	-3.6480	2.9801
AD	Right.VI	-0.0668	-0.0085	0.8640	-2.0922	1.9113
HC	Right.VI	0.0820	-0.0165	1.0419	-2.6692	3.3797
AD	Right.VIIB	-0.0742	-0.1273	0.8113	-2.4366	1.8056
HC	Right.VIIB	0.2298	0.2908	0.8471	-2.1946	2.4171
AD	Right.VIIIA	0.2980	0.2809	0.8886	-2.8868	2.0874
HC	Right.VIIIA	0.2290	0.1383	0.9003	-1.9708	2.6845
AD	Right.VIIIB	-0.0624	-0.0234	1.0055	-3.3033	2.1434
HC	Right.VIIIB	-0.0756	-0.0490	0.8890	-2.7500	2.0806
AD	Right.X	0.3633	0.4382	0.9948	-2.8421	2.6639
HC	Right.X	0.3375	0.2386	1.0367	-2.7162	3.2078
AD	Rigt.IV	-0.1557	-0.0882	0.9444	-2.6226	2.2004
HC	Rigt.IV	0.1072	0.2311	0.9487	-2.4665	2.3505
AD	Vermis.IX	-0.0576	-0.0668	0.9061	-2.1660	2.2167
HC	Vermis.IX	0.0777	0.0960	0.8267	-2.0345	2.6290
AD	Vermis.VI	-0.1218	-0.2013	0.9911	-2.0694	2.3111
HC	Vermis.VI	-0.0191	0.0428	1.0579	-3.1575	2.4881
AD	Vermis.VII	-0.0135	-0.0312	0.8688	-2.3356	2.2136
HC	Vermis.VII	0.0068	0.0412	0.9248	-1.9439	2.6345
AD	Vermis.VIII	0.1098	0.0516	0.9989	-3.0768	3.1618
HC	Vermis.VIII	0.2462	0.2582	0.9951	-2.7148	2.6036
AD	Vermis.X	0.2352	0.2288	1.0343	-2.6012	2.6116
HC	Vermis.X	0.1481	0.1905	0.9463	-2.7288	2.3445
AD	Total_Cerebel_Vol	-0.1220	-0.0663	0.8509	-2.6614	2.1197
HC	Total_Cerebel_Vol	0.1966	0.2218	0.8916	-1.9001	2.5479

Supplementary Table 4. AUROC values of each clinical cohorts

a) ASD

Atlas	Models	p_value	AUROC
anatomical	z_variance	0.001	0.595
anatomical	z_median	0.041	0.550
anatomical	z_positive	0.438	0.502
anatomical	z_negative	0.296	0.516
anatomical	z_lobules	0.005	0.582
Task-based	z_variance	0.001	0.577
Task-based	z_median	0.134	0.524
Task-based	z_positive	0.757	0.481
Task-based	z_negative	0.001	0.559
Hierarchical	z_variance	0.015	0.554
Hierarchical	z_median	0.004	0.558
Hierarchical	z_positive	0.345	0.512
Hierarchical	z_negative	0.150	0.526
Resting-state	z_variance	0.054	0.541
Resting-state	z_median	0.224	0.519
Resting-state	z_positive	0.255	0.515
Resting-state	z_negative	0.076	0.531

b) BD

Atlas	Models	p_value	AUROC
anatomical	z_variance	0.837	0.463
anatomical	z_median	0.283	0.520
anatomical	z_positive	0.481	0.501
anatomical	z_negative	0.733	0.476
anatomical	z_lobules	0.576	0.493
Task-based	z_variance	0.687	0.480
Task-based	z_median	0.415	0.507
Task-based	z_positive	0.967	0.434
Task-based	z_negative	0.341	0.510
Hierarchical	z_variance	0.779	0.470
Hierarchical	z_median	0.685	0.480

Hierarchical	z_positive	0.076	0.548
Hierarchical	z_negative	0.134	0.539
Resting-state	z_variance	0.854	0.459
Resting-state	z_median	0.045	0.553
Resting-state	z_positive	0.298	0.517
Resting-state	z_negative	0.127	0.540

c) SZ

Atlas	Models	p_value	AUROC
anatomical	z_variance	0.035	0.567
anatomical	z_median	0.001	0.636
anatomical	z_positive	0.013	0.576
anatomical	z_negative	0.001	0.633
anatomical	z_lobules	0.008	0.597
Task-based	z_variance	0.282	0.518
Task-based	z_median	0.001	0.632
Task-based	z_positive	0.002	0.578
Task-based	z_negative	0.001	0.634
Hierarchical	z_variance	0.008	0.586
Hierarchical	z_median	0.001	0.630
Hierarchical	z_positive	0.059	0.550
Hierarchical	z_negative	0.001	0.648
Resting-state	z_variance	0.231	0.526
Resting-state	z_median	0.001	0.628
Resting-state	z_positive	0.047	0.547
Resting-state	z_negative	0.001	0.618

d) MCI

Atlas	Models	p_value	AUROC
anatomical	z_variance	0.994	0.346
anatomical	z_median	0.060	0.573
anatomical	z_positive	0.254	0.524
anatomical	z_negative	0.006	0.620
anatomical	z_lobules	0.078	0.577
Task-based	z_variance	0.938	0.395
Task-based	z_median	0.025	0.583

Task-based	z_positive	0.256	0.522
Task-based	z_negative	0.018	0.583
Hierarchical	z_variance	0.857	0.433
Hierarchical	z_median	0.062	0.574
Hierarchical	z_positive	0.130	0.552
Hierarchical	z_negative	0.157	0.541
Resting-state	z_variance	0.941	0.396
Resting-state	z_median	0.018	0.597
Resting-state	z_positive	0.493	0.497
Resting-state	z_negative	0.021	0.591

e) AD

Atlas	Models	p_value	AUROC
anatomical	z_variance	0.836	0.433
anatomical	z_median	0.049	0.574
anatomical	z_positive	0.285	0.523
anatomical	z_negative	0.033	0.586
anatomical	z_lobules	0.014	0.612
Task-based	z_variance	0.849	0.426
Task-based	z_median	0.026	0.580
Task-based	z_positive	0.146	0.537
Task-based	z_negative	0.008	0.587
Hierarchical	z_variance	0.970	0.389
Hierarchical	z_median	0.036	0.578
Hierarchical	z_positive	0.285	0.526
Hierarchical	z_negative	0.014	0.600
Resting-state	z_variance	0.929	0.413
Resting-state	z_median	0.068	0.573
Resting-state	z_positive	0.147	0.542
Resting-state	z_negative	0.408	0.511

Supplementary Table 5. Feature Importance of ASD

a) Feature Importance of ASD in lobules

Features	z_lobules_Importance
Corpus.Medullare	0.433
Left.Crus.I	0.395
Left.Crus.II	0.643
Left.I.III	0.091
Left.IV	0.058
Left.IX	0.260
Left.V	0.106
Left.VI	0.352
Left.VIIB	0.068
Left.VIIIA	0.293
Left.VIIIB	0.299
Left.X	0.579
Right.Crus.I	0.289
Right.Crus.II	0.247
Right.I.III	0.123
Right.IX	0.162
Right.V	0.460
Right.VI	1.000
Right.VIIB	0.398
Right.VIIIA	0.070
Right.VIIIB	0.408
Right.X	0.289
Rigt.IV	0.143
Vermis.IX	0.429
Vermis.VI	0.057
Vermis.VII	0.000
Vermis.VIII	0.128
Vermis.X	0.512

b) Feature Importance of ASD in anatomical atlas

Features	z_variance_Importance	z_median_Importance	z_positive_Importance	z_negative_Importance
Left.I.III	0.098	0.448	0.604	0.604
Right.I.III	0.414	0.087	0.210	0.201
Left.IV	0.328	0.090	0.076	0.400

Right.IV	0.048	0.182	0.110	0.157
Left.V	0.076	0.164	0.060	0.767
Right.V	0.143	0.056	0.035	0.625
Vermis.VI	0.224	0.184	0.953	0.169
Left.VI	0.197	0.052	0.113	0.199
Right.VI	0.420	0.048	0.000	0.163
Vermis.VII	0.079	0.225	0.504	0.328
Left.Crus.I	0.134	0.033	0.079	0.186
Left.Crus.II	0.865	0.136	0.340	0.489
Left.VIIB	1.000	0.082	0.132	0.000
Right.Crus.I	0.134	0.010	0.137	0.083
Right.Crus.II	0.197	0.000	0.462	0.735
Right.VIIB	0.231	0.079	0.051	0.216
Vermis.VIII	0.202	0.087	0.265	0.860
Left.VIIIA	0.343	0.441	0.574	0.066
Left.VIIIB	0.251	0.157	0.175	0.114
Right.VIIIA	0.601	0.045	0.129	0.593
Right.VIIIIA	0.417	0.065	0.282	0.239
Vermis.IX	0.203	0.002	0.624	0.860
Left.IX	0.056	0.171	0.456	0.318
Right.IX	0.111	0.135	0.257	1.000
Vermis.X	0.000	1.000	0.407	0.157
Left.X	0.328	0.561	1.000	0.413
Right.X	0.241	0.066	0.381	0.663

c) Feature Importance of ASD in task-based atlas

Features	z_variance_Importance	z_median_Importance	z_positive_Importance	z_negative_Importance
1: Left-hand presses/ motor planning/ interference resolution	0.301	0.000	0.424	0.000
2: Right-hand presses/ motor planning/ divided attention	0.834	0.253	0.129	0.411
3: Saccades/visual working memory/visual letter recognition	0.040	0.187	0.579	0.296
4: Action Observation/divided attention/motor planning	0.000	0.000	0.227	0.259

5: Divided attention/active maintenance/mental arithmetic	0.301	0.154	0.698	0.203
6: Divided attention/verbal fluency/active maintenance	0.040	0.054	0.680	0.173
7: Narrative/ emotion processing/ language processing	1.000	0.111	0.000	0.139
8: Word comprehension/ language processing/ narrative	0.690	0.251	0.424	0.253
9: Verbal Fluency/word comprehension/mental arithmetic	0.301	0.363	1.000	1.000
10: Autobiographical recall/visual letter recognition/interference resolution	0.000	1.000	0.115	0.376

d) Feature Importance of ASD in hierarchical atlas

Features	z_variance_Importance	z_median_Importance	z_positive_Importance	z_negative_Importance
1_M1L	0.308	0.205	0.054	0.124
10_D3L	0.157	0.114	0.129	0.202
11_D4L	0.266	0.323	0.155	0.402
12_S1L	0.815	0.623	0.396	0.360
13_S2L	0.142	0.328	0.110	0.226
14_S3L	0.215	0.096	0.094	0.026
15_S4L	0.307	0.634	0.664	0.289
16_S5L	0.163	0.197	0.056	0.029
17_M1R	0.071	0.150	0.257	0.087
18_M2R	0.007	0.033	0.000	0.000
19_M3R	0.018	0.000	0.116	0.033
2_M2L	0.088	0.019	0.075	0.346
20_M4R	0.018	0.053	0.257	0.101
21_A1R	0.917	0.350	0.969	0.437
22_A2R	0.032	0.297	0.190	0.111
23_A3R	0.375	0.153	0.231	0.551

24_D1R	0.199	0.631	0.399	1.000
25_D2R	0.172	0.497	0.063	0.130
26_D3R	0.345	0.171	0.038	0.224
27_D4R	0.000	0.005	0.243	0.033
28_S1R	0.471	0.104	0.157	0.494
29_S2R	0.142	0.286	0.151	0.321
3_M3L	0.112	0.095	0.072	0.510
30_S3R	0.153	0.257	0.202	0.135
31_S4R	0.591	0.239	0.908	0.180
32_S5R	0.434	0.182	0.043	0.126
4_M$L	0.802	0.212	1.000	0.290
5_A1L	0.254	1.000	0.236	0.966
6_A2L	1.000	0.491	0.158	0.107
7_A3L	0.100	0.137	0.243	0.229
8_D1L	0.202	0.398	0.119	0.507
9_D2L	0.343	0.189	0.498	0.445

e) Feature Importance of ASD in resting-state atlas

Features	z_variance_Importance	z_median_Importance	z_positive_Importance	z_negative_Importance
1: Visual A	0.068	1.000	0.050	0.155
2: Visual B	1.000	0.920	1.000	0.317
3: Somatomotor A	0.000	0.045	0.561	0.107
4: Somatomotor B	0.056	0.222	0.048	0.173
5: Dorsal Attention A	0.994	0.284	0.986	1.000
6: Dorsal Attention B	0.507	0.683	0.234	0.052
7: Salience/Ventral Attention A	0.095	0.073	0.672	0.347
8: Salience/ Ventral Attention B	0.151	0.166	0.052	0.668
9: Limbic B	0.193	0.158	0.054	0.253
10: Limbic A	0.121	0.251	0.904	0.243
11: Control A	0.077	0.084	0.131	0.615
12 Control B	0.000	0.061	0.140	0.067
13: Control C	0.320	0.000	0.083	0.054
14: Default A	0.388	0.230	0.264	0.063
15: Default B	0.228	0.428	0.226	0.247
16: Default C	0.753	0.353	0.000	0.000
17: Temporal Parietal	0.320	0.026	0.251	0.251

Supplementary Table 6. Feature Importance of BD

a) Feature Importance of BD in lobules

Features	z_lobules_Importance
Corpus.Medullare	0.460
Left.Crus.I	0.148
Left.Crus.II	0.159
Left.I.III	0.256
Left.IV	0.228
Left.IX	0.858
Left.V	0.206
Left.VI	0.511
Left.VIIB	0.105
Left.VIIIA	0.000
Left.VIIIB	0.163
Left.X	0.388
Right.Crus.I	0.593
Right.Crus.II	0.372
Right.I.III	0.237
Right.IX	0.572
Right.V	0.000
Right.VI	1.000
Right.VIIB	0.105
Right.VIIIA	0.107
Right.VIIIB	0.234
Right.X	0.312
Rigt.IV	0.485
Vermis.IX	0.646
Vermis.VI	0.482
Vermis.VII	0.360
Vermis.VIII	0.202
Vermis.X	0.039

b) Feature Importance of BD in anatomical atlas

Features	z_variance_Importance	z_median_Importance	z_positive_Importance	z_negative_Importance
Left.I.III	0.167	0.253	0.416	0.392
Right.I.III	0.186	0.499	0.125	0.557
Left.IV	0.306	0.300	0.157	0.701
Right.IV	0.175	0.421	0.070	1.000
Left.V	0.495	0.267	0.142	0.786

Right.V	0.266	0.039	0.530	0.075
Vermis.VI	0.365	0.300	0.095	0.821
Left.VI	0.171	0.077	0.187	0.000
Right.VI	0.177	0.000	0.140	0.516
Vermis.VII	0.541	0.435	0.483	0.951
Left.Crus.I	0.783	0.060	0.223	0.167
Left.Crus.II	1.000	0.338	0.000	0.288
Left.VIIB	0.121	0.322	0.153	0.608
Right.Crus.I	0.956	0.004	0.109	0.055
Right.Crus.II	0.000	0.047	0.210	0.374
Right.VIIB	0.300	0.219	0.607	0.269
Vermis.VIII	0.582	0.631	0.501	0.987
Left.VIIIA	0.047	0.007	0.262	0.458
Left.VIIIB	0.000	0.311	0.291	0.114
Right.VIIIA	0.765	0.103	0.271	0.183
Right.VIIIIA	0.285	0.240	0.072	0.423
Vermis.IX	0.110	0.441	0.248	0.201
Left.IX	0.318	0.456	0.420	0.548
Right.IX	0.752	0.060	0.096	0.053
Vermis.X	0.403	0.273	0.534	0.352
Left.X	0.154	1.000	1.000	0.183
Right.X	0.470	0.234	0.206	0.544

c) Feature Importance of BD in task-based atlas

Features	z_variance_Importance	z_median_Importance	z_positive_Importance	z_negative_Importance
1: Left-hand presses/ motor planning/ interference resolution	1.000	0.002	0.275	0.296
2: Right-hand presses/ motor planning/ divided attention	0.008	0.358	0.412	0.523
3: Saccades/visual working memory/visual letter recognition	0.532	0.428	0.384	0.000
4: Action Observation/divided attention/motor planning	0.048	0.000	0.583	0.432
5: Divided attention/active maintenance/mental arithmetic	0.052	0.147	0.118	0.031

6: Divided attention/verbal fluency/active maintenance	0.407	0.301	0.000	0.383
7: Narrative/ emotion processing/ language processing	0.008	0.183	0.654	0.129
8: Word comprehension/ language processing/ narrative	0.000	0.404	0.327	0.822
9: Verbal Fluency/word comprehension/mental arithmetic	0.005	1.000	1.000	0.254
10: Autobiographical recall/visual letter recognition/interference resolution	0.048	0.064	0.275	1.000

d) Feature Importance of BD in hierarchical atlas

Features	z_variance_Importance	z_median_Importance	z_positive_Importance	z_negative_Importance
1_M1L	0.593	0.607	1.000	1.000
10_D3L	0.270	0.008	0.785	0.893
11_D4L	0.270	0.266	0.723	0.878
12_S1L	0.370	0.730	0.677	0.782
13_S2L	0.630	0.080	0.518	0.762
14_S3L	1.000	0.067	0.429	0.748
15_S4L	0.413	0.282	0.374	0.386
16_S5L	0.113	0.173	0.323	0.383
17_M1R	0.069	0.135	0.318	0.383
18_M2R	0.612	0.159	0.309	0.377
19_M3R	0.270	0.101	0.285	0.346
2_M2L	0.222	0.173	0.278	0.308
20_M4R	0.557	0.133	0.274	0.294
21_A1R	0.168	0.175	0.259	0.287
22_A2R	0.328	0.353	0.255	0.285
23_A3R	0.223	0.126	0.252	0.275
24_D1R	0.193	0.241	0.241	0.268
25_D2R	0.307	0.289	0.213	0.223
26_D3R	0.231	0.000	0.206	0.205
27_D4R	0.612	0.058	0.203	0.203

28_S1R	0.515	1.000	0.179	0.192
29_S2R	0.039	0.108	0.176	0.185
3_M3L	0.201	0.042	0.098	0.154
30_S3R	0.201	0.065	0.083	0.138
31_S4R	0.631	0.261	0.077	0.129
32_S5R	0.530	0.264	0.073	0.128
4_M$L	0.167	0.226	0.066	0.121
5_A1L	0.126	0.347	0.063	0.105
6_A2L	0.540	0.190	0.037	0.102
7_A3L	0.566	0.209	0.036	0.081
8_D1L	0.000	0.437	0.013	0.073
9_D2L	0.069	0.186	0.000	0.000

e) Feature Importance of BD in resting-state atlas

Features	z_variance_Importance	z_median_Importance	z_positive_Importance	z_negative_Importance
1: Visual A	0.042	0.410	0.247	0.247
2: Visual B	0.192	0.069	0.526	0.752
3: Somatomotor A	0.542	0.155	0.111	0.076
4: Somatomotor B	0.000	0.012	0.085	0.170
5: Dorsal Attention A	0.217	0.269	0.405	0.079
6: Dorsal Attention B	0.218	0.019	0.113	0.069
7: Salience/Ventral Attention A	0.646	0.030	0.049	0.134
8: Salience/ Ventral Attention B	0.221	0.147	0.514	0.319
9: Limbic B	0.552	0.018	0.186	0.274
10: Limbic A	0.306	1.000	0.188	0.218
11: Control A	0.127	0.007	0.073	1.000
12 Control B	0.787	0.189	0.066	0.006
13: Control C	0.096	0.058	0.000	0.000
14: Default A	0.216	0.200	0.710	0.190
15: Default B	0.238	0.038	1.000	0.101
16: Default C	1.000	0.000	0.241	0.158
17: Temporal Parietal	0.402	0.207	0.788	0.201

Supplementary Table 7. Feature Importance of SZ

a) Feature Importance of SZ in lobules

Features	z_lobules_Importance
Corpus.Medullare	0.195
Left.Crus.I	0.836
Left.Crus.II	0.129
Left.I.III	1.000
Left.IV	0.454
Left.IX	0.249
Left.V	0.256
Left.VI	0.595
Left.VIIB	0.779
Left.VIIIA	0.078
Left.VIIIB	0.219
Left.X	0.187
Right.Crus.I	0.000
Right.Crus.II	0.307
Right.I.III	0.519
Right.IX	0.706
Right.V	0.203
Right.VI	0.625
Right.VIIB	0.808
Right.VIIIA	0.135
Right.VIIIB	0.297
Right.X	0.063
Rigt.IV	0.150
Vermis.IX	0.821
Vermis.VI	0.356
Vermis.VII	0.307
Vermis.VIII	0.160
Vermis.X	0.450

b) Feature Importance of SZ in anatomical atlas

Features	z_variance_Importance	z_median_Importance	z_positive_Importance	z_negative_Importance
Left.I.III	0.176	0.407	0.393	0.325
Right.I.III	0.799	1.000	0.229	0.303
Left.IV	0.009	0.061	0.232	0.918
Right.IV	0.749	0.127	0.122	0.224
Left.V	0.144	0.465	0.650	0.267

Right.V	1.000	0.288	0.300	0.244
Vermis.VI	0.318	0.158	0.324	0.575
Left.VI	0.128	0.078	0.305	0.006
Right.VI	0.274	0.114	0.043	0.212
Vermis.VII	0.341	0.192	0.121	0.022
Left.Crus.I	0.694	0.080	0.071	0.080
Left.Crus.II	0.248	0.000	0.000	0.082
Left.VIIB	0.325	0.056	0.228	0.192
Right.Crus.I	0.000	0.001	0.074	0.000
Right.Crus.II	0.232	0.072	0.070	0.423
Right.VIIB	0.490	0.495	0.131	0.138
Vermis.VIII	0.550	0.716	0.224	0.467
Left.VIIIA	0.492	0.165	0.183	0.335
Left.VIIIB	0.009	0.608	0.230	0.399
Right.VIIIA	0.451	0.190	0.279	0.497
Right.VIIIIA	0.578	0.076	0.190	0.210
Vermis.IX	0.198	0.128	0.430	1.000
Left.IX	0.492	0.246	0.144	0.483
Right.IX	0.511	0.073	1.000	0.110
Vermis.X	0.466	0.277	0.305	0.315
Left.X	0.393	0.470	0.539	0.210
Right.X	0.049	0.494	0.707	0.860

c) Feature Importance of SZ in task based atlas

Features	z_variance_Importance	z_median_Importance	z_positive_Importance	z_negative_Importance
1: Left-hand presses/ motor planning/ interference resolution	0.075	0.533	0.186	0.400
2: Right-hand presses/ motor planning/ divided attention	0.719	0.742	0.000	0.230
3: Saccades/visual working memory/visual letter recognition	0.522	0.184	0.189	0.558
4: Action Observation/divided attention/motor planning	0.297	0.356	0.262	0.000
5: Divided attention/active maintenance/mental arithmetic	0.522	1.000	0.049	0.067

6: Divided attention/verbal fluency/active maintenance	0.297	0.056	0.108	0.570
7: Narrative/ emotion processing/ language processing	1.000	0.124	0.201	0.600
8: Word comprehension/ language processing/ narrative	0.000	0.324	0.503	0.352
9: Verbal Fluency/word comprehension/mental arithmetic	0.042	0.000	1.000	1.000
10: Autobiographical recall/visual letter recognition/interference resolution	0.042	0.007	0.273	0.885

d) Feature Importance of SZ in hierarchical atlas

Features	z_variance_Importance	z_median_Importance	z_positive_Importance	z_negative_Importance
1_M1L	0.216	0.065	0.111	0.367
10_D3L	0.000	0.000	0.182	0.000
11_D4L	0.087	0.395	0.123	0.139
12_S1L	0.048	1.000	0.208	1.000
13_S2L	0.423	0.095	0.101	0.057
14_S3L	0.216	0.164	0.048	0.092
15_S4L	0.227	0.244	0.308	0.256
16_S5L	0.082	0.494	0.340	0.544
17_M1R	0.317	0.281	1.000	0.221
18_M2R	0.426	0.226	0.067	0.188
19_M3R	0.107	0.012	0.025	0.108
2_M2L	0.048	0.212	0.091	0.053
20_M4R	0.109	0.713	0.126	0.119
21_A1R	0.039	0.278	0.104	0.203
22_A2R	0.625	0.299	0.235	0.135
23_A3R	0.617	0.406	0.119	0.233
24_D1R	0.130	0.634	0.387	0.524
25_D2R	0.145	0.170	0.481	0.095
26_D3R	0.163	0.438	0.000	0.060

27_D4R	0.243	0.159	0.074	0.056
28_S1R	0.093	0.176	0.123	0.147
29_S2R	0.078	0.032	0.230	0.359
3_M3L	0.243	0.105	0.246	0.095
30_S3R	0.216	0.215	0.285	0.042
31_S4R	0.094	0.339	0.652	0.246
32_S5R	0.039	0.169	0.588	0.266
4_M$L	0.074	0.119	0.106	0.080
5_A1L	0.287	0.891	0.213	0.517
6_A2L	0.163	0.338	0.185	0.433
7_A3L	0.214	0.258	0.109	0.677
8_D1L	1.000	0.915	0.454	0.738
9_D2L	0.059	0.200	0.241	0.093

e) Feature Importance of SZ in resting-state atlas

Features	z_variance_Importance	z_median_Importance	z_positive_Importance	z_negative_Importance
1: Visual A	0.178	0.187	0.333	0.108
2: Visual B	0.503	1.000	0.998	0.112
3: Somatomotor A	0.631	0.156	0.154	0.332
4: Somatomotor B	1.000	0.105	1.000	0.147
5: Dorsal Attention A	0.202	0.328	0.934	1.000
6: Dorsal Attention B	0.372	0.052	0.000	0.137
7: Salience/Ventral Attention A	0.540	0.000	0.499	0.119
8: Salience/ Ventral Attention B	0.768	0.077	0.156	0.000
9: Limbic B	0.541	0.270	0.586	0.016
10: Limbic A	0.468	0.821	0.513	0.475
11: Control A	0.653	0.163	0.215	0.036
12 Control B	0.927	0.008	0.199	0.069
13: Control C	0.000	0.084	0.104	0.158
14: Default A	0.141	0.438	0.208	0.228
15: Default B	0.339	0.342	0.541	0.108
16: Default C	0.000	0.127	0.073	0.057
17: Temporal Parietal	0.548	0.039	0.426	0.002

Supplementary Table 8. Feature Importance of MCI

a) Feature Importance of MCI in lobules

Features	z_lobules_Importance
Corpus.Medullare	0.628
Left.Crus.I	0.132
Left.Crus.II	0.561
Left.I.III	0.274
Left.IV	1.000
Left.IX	0.767
Left.V	0.155
Left.VI	0.399
Left.VIIB	0.261
Left.VIIIA	0.093
Left.VIIIB	0.017
Left.X	0.072
Right.Crus.I	0.332
Right.Crus.II	0.194
Right.I.III	0.671
Right.IX	0.264
Right.V	0.600
Right.VI	0.716
Right.VIIB	0.133
Right.VIIIA	0.151
Right.VIIIB	0.200
Right.X	0.172
Rigt.IV	0.317
Vermis.IX	0.079
Vermis.VI	0.356
Vermis.VII	0.000
Vermis.VIII	0.013
Vermis.X	0.100

b) Feature Importance of MCI in anatomical atlas

Features	z_variance_Importance	z_median_Importance	z_positive_Importance	z_negative_Importance
Left.I.III	0.013	0.094	0.346	0.188
Right.I.III	0.237	0.361	0.164	0.442
Left.IV	0.721	0.048	0.098	0.281
Right.IV	0.559	0.162	0.030	0.291

Left.V	0.245	0.244	0.100	0.145
Right.V	0.294	0.448	0.060	0.071
Vermis.VI	0.166	0.277	0.832	0.426
Left.VI	0.013	0.008	0.000	0.048
Right.VI	0.258	0.061	0.076	0.052
Vermis.VII	0.166	0.234	1.000	0.475
Left.Crus.I	0.019	0.050	0.134	0.000
Left.Crus.II	0.122	0.369	0.168	0.296
Left.VIIB	0.166	0.034	0.009	0.021
Right.Crus.I	0.013	0.000	0.008	0.012
Right.Crus.II	0.508	0.044	0.021	0.124
Right.VIIB	0.000	0.257	0.276	0.071
Vermis.VIII	0.243	0.238	0.543	0.541
Left.VIIIA	0.079	0.086	0.008	0.066
Left.VIIIB	0.196	0.414	0.060	0.117
Right.VIIIA	0.212	0.151	0.002	0.223
Right.VIIIIA	0.228	0.179	0.295	0.050
Vermis.IX	0.352	1.000	0.178	0.777
Left.IX	0.046	0.174	0.081	0.172
Right.IX	0.245	0.179	0.176	0.193
Vermis.X	0.276	0.212	0.238	0.620
Left.X	1.000	0.448	0.745	0.372
Right.X	0.314	0.704	0.577	1.000

c) Feature Importance of MCI in task-based atlas

Features	z_variance_Importance	z_median_Importance	z_positive_Importance	z_negative_Importance
1: Left-hand presses/ motor planning/ interference resolution	0.012	0.087	0.270	0.000
2: Right-hand presses/ motor planning/ divided attention	0.233	0.000	0.000	0.786
3: Saccades/visual working memory/visual letter recognition	0.330	0.508	0.198	0.491
4: Action Observation/divided attention/motor planning	0.000	0.377	0.365	0.077
5: Divided attention/active	1.000	0.297	0.108	0.682

maintenance/mental arithmetic				
6: Divided attention/verbal fluency/active maintenance	0.243	0.222	0.059	0.945
7: Narrative/ emotion processing/ language processing	0.732	1.000	0.117	0.545
8: Word comprehension/ language processing/ narrative	0.791	0.306	1.000	0.659
9: Verbal Fluency/word comprehension/mental arithmetic	0.012	0.512	0.730	0.168
10: Autobiographical recall/visual letter recognition/interference resolution	0.945	0.173	0.315	1.000

d) Feature Importance of MCI in hierarchical atlas

Features	z_variance_Importance	z_median_Importance	z_positive_Importance	z_negative_Importance
1_M1L	0.288	0.720	0.077	0.230
10_D3L	0.036	0.227	0.166	0.026
11_D4L	0.193	0.300	0.074	0.105
12_S1L	0.552	0.404	0.172	0.000
13_S2L	0.256	0.214	0.086	0.234
14_S3L	0.000	0.215	0.155	0.108
15_S4L	0.237	1.000	0.294	0.214
16_S5L	0.303	0.165	0.062	0.035
17_M1R	1.000	0.180	0.404	0.216
18_M2R	0.170	0.099	0.176	0.071
19_M3R	0.511	0.193	0.018	0.015
2_M2L	0.122	0.062	0.023	0.039
20_M4R	0.350	0.177	0.383	0.158
21_A1R	0.135	0.407	0.268	1.000
22_A2R	0.764	0.385	0.439	0.176
23_A3R	0.568	0.433	0.000	0.059
24_D1R	0.547	0.462	0.155	0.239

25_D2R	0.068	0.178	0.231	0.079
26_D3R	0.071	0.007	0.036	0.179
27_D4R	0.372	0.051	0.072	0.005
28_S1R	0.028	0.631	0.158	0.087
29_S2R	0.154	0.000	0.117	0.257
3_M3L	0.427	0.072	0.101	0.046
30_S3R	0.328	0.011	0.107	0.085
31_S4R	0.578	0.953	1.000	0.541
32_S5R	0.028	0.561	0.031	0.087
4_M$L	0.215	0.725	0.113	0.044
5_A1L	0.785	0.127	0.458	0.028
6_A2L	0.224	0.398	0.371	0.093
7_A3L	0.307	0.799	0.967	0.273
8_D1L	0.956	0.393	0.396	0.728
9_D2L	0.097	0.234	0.118	0.051

e) Feature Importance of MCI in resting-state atlas

Features	z_variance_Importance	z_median_Importance	z_positive_Importance	z_negative_Importance
1: Visual A	0.012	0.155	0.158	0.328
2: Visual B	0.644	0.141	0.351	0.968
3: Somatomotor A	0.098	0.129	0.000	0.133
4: Somatomotor B	0.098	0.174	0.326	0.083
5: Dorsal Attention A	0.556	0.331	1.000	0.159
6: Dorsal Attention B	0.243	0.013	0.122	0.107
7: Salience/Ventral Attention A	0.214	0.000	0.213	0.133
8: Salience/ Ventral Attention B	0.036	0.024	0.051	0.000
9: Limbic B	0.475	0.140	0.138	0.398
10: Limbic A	0.328	0.572	0.317	0.272
11: Control A	1.000	0.052	0.333	0.225
12 Control B	0.259	0.067	0.358	0.077
13: Control C	0.000	0.037	0.000	0.087
14: Default A	0.173	1.000	0.376	1.000
15: Default B	0.397	0.496	0.204	0.737
16: Default C	0.243	0.049	0.186	0.078
17: Temporal Parietal	0.243	0.067	0.021	0.059

Supplementary Table 9. Feature Importance of AD

a) Feature Importance of AD in lobules

Features	z_lobules_Importance
Corpus.Medullare	0.484
Left.Crus.I	0.199
Left.Crus.II	0.644
Left.I.III	0.966
Left.IV	0.045
Left.IX	0.145
Left.V	0.051
Left.VI	0.255
Left.VIIB	0.685
Left.VIIIA	1.000
Left.VIIIB	0.290
Left.X	0.242
Right.Crus.I	0.566
Right.Crus.II	0.366
Right.I.III	0.487
Right.IX	0.190
Right.V	0.204
Right.VI	0.090
Right.VIIB	0.949
Right.VIIIA	0.667
Right.VIIIB	0.000
Right.X	0.155
Rigt.IV	0.801
Vermis.IX	0.021
Vermis.VI	0.150
Vermis.VII	0.122
Vermis.VIII	0.007
Vermis.X	0.475

b) Feature Importance of AD in anatomical atlas

Features	z_variance_Importance	z_median_Importance	z_positive_Importance	z_negative_Importance
Left.I.III	1.000	0.842	0.081	1.000
Right.I.III	0.509	0.142	0.651	0.177
Left.IV	0.117	0.307	0.104	0.013
Right.IV	0.253	0.865	0.558	0.393
Left.V	0.107	0.847	0.102	0.094

Right.V	0.164	0.872	0.356	0.059
Vermis.VI	0.964	0.512	0.207	0.226
Left.VI	0.000	0.067	0.155	0.206
Right.VI	0.000	0.327	0.164	0.069
Vermis.VII	0.347	0.407	0.451	0.328
Left.Crus.I	0.239	0.022	0.041	0.006
Left.Crus.II	0.008	0.516	0.066	0.001
Left.VIIB	0.000	0.053	0.047	0.256
Right.Crus.I	0.245	0.000	0.021	0.078
Right.Crus.II	0.350	0.156	0.193	0.212
Right.VIIB	0.037	0.102	0.000	0.000
Vermis.VIII	0.433	0.181	0.057	0.326
Left.VIIIA	0.034	0.634	0.660	0.041
Left.VIIIB	0.035	0.264	0.064	0.081
Right.VIIIA	0.034	0.414	0.271	0.396
Right.VIIIIA	0.227	0.717	0.192	0.027
Vermis.IX	0.417	0.930	0.770	0.379
Left.IX	0.334	1.000	0.083	0.115
Right.IX	0.837	0.489	0.159	0.211
Vermis.X	0.379	0.517	1.000	0.677
Left.X	0.209	0.675	0.124	0.057
Right.X	0.263	0.684	0.416	0.490

c) Feature Importance of AD in task-based atlas

Features	z_variance_Importance	z_median_Importance	z_positive_Importance	z_negative_Importance
1: Left-hand presses/ motor planning/ interference resolution	0.636	0.000	0.439	0.395
2: Right-hand presses/ motor planning/ divided attention	0.912	0.304	0.004	0.340
3: Saccades/visual working memory/visual letter recognition	0.000	0.466	1.000	0.090
4: Action Observation/divided attention/motor planning	0.362	1.000	0.067	0.077
5: Divided attention/active	0.360	0.366	0.028	0.525

maintenance/mental arithmetic				
6: Divided attention/verbal fluency/active maintenance	0.775	0.215	0.000	0.040
7: Narrative/ emotion processing/ language processing	0.420	0.942	0.063	0.469
8: Word comprehension/ language processing/ narrative	0.692	0.734	0.269	0.315
9: Verbal Fluency/word comprehension/mental arithmetic	0.635	0.690	0.597	0.000
10: Autobiographical recall/visual letter recognition/interference resolution	1.000	0.175	0.028	1.000

d) Feature Importance of AD in hierarchical atlas

Features	z_variance_Importance	z_median_Importance	z_positive_Importance	z_negative_Importance
1_M1L	0.213	0.184	0.054	0.091
10_D3L	0.035	0.005	0.129	0.295
11_D4L	0.198	0.048	0.155	0.642
12_S1L	0.605	0.066	0.396	0.250
13_S2L	0.284	0.070	0.110	0.111
14_S3L	0.056	0.044	0.094	0.000
15_S4L	1.000	0.118	0.664	0.473
16_S5L	0.869	0.024	0.056	0.301
17_M1R	0.577	0.085	0.257	0.654
18_M2R	0.607	0.034	0.000	0.149
19_M3R	0.332	0.205	0.116	0.171
2_M2L	0.000	0.069	0.075	0.091
20_M4R	0.291	0.058	0.257	0.226
21_A1R	0.035	0.583	0.969	0.260
22_A2R	0.354	0.055	0.190	0.146
23_A3R	0.429	0.213	0.231	0.057
24_D1R	0.035	0.168	0.399	0.283

25_D2R	0.035	0.184	0.063	0.235
26_D3R	0.056	0.021	0.038	0.225
27_D4R	0.000	0.136	0.243	0.094
28_S1R	0.283	0.297	0.157	0.014
29_S2R	0.000	0.154	0.151	0.354
3_M3L	0.000	0.206	0.072	0.104
30_S3R	0.000	0.207	0.202	0.345
31_S4R	0.735	0.149	0.908	1.000
32_S5R	0.383	0.296	0.043	0.478
4_M$L	0.147	0.085	1.000	0.105
5_A1L	0.560	0.226	0.236	0.365
6_A2L	0.147	0.122	0.158	0.290
7_A3L	0.428	0.232	0.243	0.543
8_D1L	0.318	1.000	0.119	0.201
9_D2L	0.397	0.000	0.498	0.136

e) Feature Importance of AD in resting-state atlas

Features	z_variance_Importance	z_median_Importance	z_positive_Importance	z_negative_Importance
1: Visual A	0.317	0.642	0.263	0.306
2: Visual B	0.027	1.000	0.206	0.394
3: Somatomotor A	0.556	0.210	0.161	0.620
4: Somatomotor B	0.705	0.000	0.153	0.083
5: Dorsal Attention A	0.281	0.578	0.434	0.770
6: Dorsal Attention B	0.166	0.015	0.089	0.382
7: Salience/Ventral Attention A	0.151	0.071	0.030	0.000
8: Salience/ Ventral Attention B	0.584	0.308	0.032	0.134
9: Limbic B	0.251	0.237	0.158	0.327
10: Limbic A	0.043	0.449	0.330	1.000
11: Control A	0.921	0.623	0.309	0.382
12 Control B	0.088	0.197	0.048	0.841
13: Control C	0.076	0.256	0.000	0.389
14: Default A	0.671	0.334	1.000	0.311
15: Default B	1.000	0.265	0.437	0.931
16: Default C	0.151	0.307	0.039	0.433
17: Temporal Parietal	0.000	0.473	0.009	0.196

References

1. Kim M, Leonardsen E, Rutherford S, Selbæk G, Persson K, Steen NE, *et al.* (2024): Mapping cerebellar anatomical heterogeneity in mental and neurological illnesses. *Nat Mental Health* 1–12.
2. Han S, Carass A, He Y, Prince JL (2020): Automatic cerebellum anatomical parcellation using U-Net with locally constrained optimization. *NeuroImage* 218: 116819.
3. Sylabs (2021): *Singularity*. https://sylabs.io/singularity.
4. King M, Hernandez-Castillo CR, Poldrack RA, Ivry RB, Diedrichsen J (2019): Functional boundaries in the human cerebellum revealed by a multi-domain task battery. *Nat Neurosci* 22: 1371–1378.
5. Nettekoven C, Zhi D, Shahshahani L, Pinho AL, Saadon-Grosman N, Buckner RL, Diedrichsen J (2024): A hierarchical atlas of the human cerebellum for functional precision mapping. *bioRxiv* 2023.09.14.557689.
6. Buckner RL, Krienen FM, Castellanos A, Diaz JC, Yeo BTT (2011): The organization of the human cerebellum estimated by intrinsic functional connectivity. *Journal of Neurophysiology* 106: 2322–2345.
7. Yeo BTT, Krienen FM, Sepulcre J, Sabuncu MR, Lashkari D, Hollinshead M, *et al.* (2011): The organization of the human cerebral cortex estimated by intrinsic functional connectivity. *Journal of Neurophysiology* 106: 1125–1165.
8. Di Martino A, Yan C-G, Li Q, Denio E, Castellanos FX, Alaerts K, *et al.* (2014): The autism brain imaging data exchange: towards a large-scale evaluation of the intrinsic brain architecture in autism [no. 6]. *Mol Psychiatry* 19: 659–667.

9. Di Martino A, O'Connor D, Chen B, Alaerts K, Anderson JS, Assaf M, *et al.* (2017): Enhancing studies of the connectome in autism using the autism brain imaging data exchange II [no. 1]. *Sci Data* 4: 170010.

10. Ellis KA, Bush AI, Darby D, De Fazio D, Foster J, Hudson P, *et al.* (2009): The Australian Imaging, Biomarkers and Lifestyle (AIBL) study of aging: methodology and baseline characteristics of 1112 individuals recruited for a longitudinal study of Alzheimer's disease. *Int Psychogeriatr* 21: 672–687.

11. Córdova-Palomera A, Kaufmann T, Persson K, Alnæs D, Doan NT, Moberget T, *et al.* (2017): Disrupted global metastability and static and dynamic brain connectivity across individuals in the Alzheimer's disease continuum. *Sci Rep* 7: 40268.

12. Doan NT, Engvig A, Persson K, Alnæs D, Kaufmann T, Rokicki J, *et al.* (2017): Dissociable diffusion MRI patterns of white matter microstructure and connectivity in Alzheimer's disease spectrum. *Sci Rep* 7: 45131.

13. Dørum ES, Alnæs D, Kaufmann T, Richard G, Lund MJ, Tønnesen S, *et al.* (2016): Age-related differences in brain network activation and co-activation during multiple object tracking. *Brain Behav* 6: e00533.

14. Doan NT, Kaufmann T, Bettella F, Jørgensen KN, Brandt CL, Moberget T, *et al.* (2017): Distinct multivariate brain morphological patterns and their added predictive value with cognitive and polygenic risk scores in mental disorders. *NeuroImage: Clinical* 15: 719–731.

15. Rimol LM, Hartberg CB, Nesvåg R, Fennema-Notestine C, Hagler DJ, Pung CJ, *et al.* (2010): Cortical thickness and subcortical volumes in schizophrenia and bipolar disorder. *Biol Psychiatry* 68: 41–50.

16. Tønnesen S, Kaufmann T, Doan NT, Alnæs D, Córdova-Palomera A, Meer D van der, *et al.* (2018): White matter aberrations and age-related trajectories in patients with schizophrenia and bipolar disorder revealed by diffusion tensor imaging [no. 1]. *Sci Rep* 8: 14129.
